# Extracellular metallothionein as a therapeutic target in the early progression of type 1 diabetes

**DOI:** 10.1016/j.cstres.2024.03.005

**Published:** 2024-03-13

**Authors:** Clare K. Melchiorre, Matthew D. Lynes, Sadikshya Bhandari, Sheng-Chiang Su, Christian M. Potts, Amy V. Thees, Carol E. Norris, Lucy Liaw, Yu-Hua Tseng, Michael A. Lynes

**Affiliations:** 1 Department of Molecular and Cell Biology, University of Connecticut, Storrs, CT, USA; 2 Center for Molecular Medicine, MaineHealth Institute for Research, Scarborough, ME, USA; 3 Section on Integrative Physiology and Metabolism, Joslin Diabetes Center, Harvard Medical School, Boston, MA, USA; 4 Division of Endocrinology and Metabolism, Department of Internal Medicine, Tri-Service General Hospital, National Defense Medical Center, Taipei, Taiwan

**Keywords:** Metallothionein, Type 1 diabetes, Chemotaxis, Therapeutic, Stress response

## Abstract

Type 1 diabetes (T1D) is characterized by lymphocyte infiltration into the pancreatic islets of Langerhans, leading to the destruction of insulin-producing beta cells and uncontrolled hyperglycemia. In the nonobese diabetic (NOD) murine model of T1D, the onset of this infiltration starts several weeks before glucose dysregulation and overt diabetes. Recruitment of immune cells to the islets is mediated by several chemotactic cytokines, including CXCL10, while other cytokines, including SDF-1α, can confer protective effects. Global gene expression studies of the pancreas from prediabetic NOD mice and single-cell sequence analysis of human islets from prediabetic, autoantibody-positive patients showed an increased expression of metallothionein (MT), a small molecular weight, cysteine-rich metal-binding stress response protein. We have shown that beta cells can release MT into the extracellular environment, which can subsequently enhance the chemotactic response of Th1 cells to CXCL10 and interfere with the chemotactic response of Th2 cells to SDF-1α. These effects can be blocked *in vitro* with a monoclonal anti-MT antibody, clone UC1MT. When administered to NOD mice before the onset of diabetes, UC1MT significantly reduces the development of T1D. Manipulation of extracellular MT may be an important approach to preserving beta cell function and preventing the development of T1D.

## Introduction

Type 1 diabetes (T1D) is an autoimmune disease that results from the destruction of insulin-producing beta cells in the pancreas by infiltrating immune cells, including CD4+ and CD8+ T cells and macrophages.^1^ About 1.6 million Americans are currently diagnosed with T1D, and there are 64,000 new diagnoses each year, 15% of whom are children. There has been a sharp increase in the incidence of T1D in pediatric populations in recent years, with steeper increases observed in racial/ethnic minority youths.^2^ T1D is currently uncurable and requires lifelong dependence on insulin therapy, with complications that can include retinopathy, neuropathy, diabetic kidney disease, and cardiovascular disease.

Nonobese diabetic (NOD) mice, the most common animal model of T1D, express anti-insulin autoantibodies (IAA) as early as 3 weeks of age with a peak seen between 8 and 16 weeks of age.^3^ IAA expression is strongly correlated with the development of T1D in both mouse and human courses of disease. Concurrent with IAA expression, there is a marked increase in immune infiltrate surrounding the pancreatic islets beginning at 4 weeks of age, and insulitis at around 10 weeks of age.^4^ Crucial to the recruitment of these cells and ensuing beta cell destruction are chemokines including CCL5, CX3CL1, and CXCL10, which are detectable in the serum of prediabetic and newly diagnosed T1D individuals.^5^ CXCL10 overexpression is associated with accelerated development of T1D,^6^ and blocking the interaction of CXCL10 with its cognate receptor CXCR3 using neutralizing antibodies reduces the incidence of T1D in mice.^7^ Other cytokines, including SDF-1α, seem to play a protective role in diabetes by recruiting Th2 cells and promoting pancreatic beta cell survival.^8^

Metallothioneins (MTs) are small molecular weight, cysteine-rich metal-binding stress response proteins that are readily inducible in many tissues upon exposure to a variety of stressors, including divalent heavy metal cations, reactive oxygen species, and irradiation.^9^ MTs can also be induced by several inflammatory mediators such as glucocorticoids, bacterial endotoxin, and acute phase cytokines such as IL-1, IL-6, and TNFα.^9^ Of the four isoforms of mammalian MT, MT1 (including functional genes *MT1A, MT1B, MT1E, MT1F, MT1G, MT1H, MT1M*, and *MT1X)* and MT2 are expressed in most tissues, while MT3 is preferentially expressed in brain tissues, and MT4 is mostly expressed in stratified squamous epithelium.^10^ MT can function as a reservoir of essential metals such as zinc and copper and can sequester toxicants including heavy metals and free radicals.^11^ In addition to aiding in the maintenance of physiological homeostasis, MT has several important immunomodulatory functions. Exogenous MT has a suppressive effect on T-cell-dependent humoral immunity,^12^ while splenocytes from mice with disrupted *Mt* genes exhibit enhanced T-dependent humoral immunity.^13^ MT can also bind to the surface of leukocytes, stimulate lymphocyte proliferation, and enhance the capacity of naïve B lymphocytes to differentiate into plasma cells.^14^, ^15^, ^16^, ^17^

MTs can be found in many extracellular spaces, including exocrine pancreatic secretions and adipocyte cell culture supernatant, suggesting that they may be selectively released from these sources.^9^, ^18^ This pool of extracellular MT can act as a chemotactic signal and has been implicated in several autoimmune and chronic or acute inflammatory diseases, including inflammatory bowel diseases (IBD), acetaminophen-induced liver injury (AILI), rheumatoid arthritis, multiple sclerosis, and sepsis.^19^, ^20^, ^21^, ^22^, ^23^ When UC1MT (anti-MT monoclonal antibody) is administered in animal models of IBD^24^ and AILI,^20^ there is a marked reduction in inflammation and an improvement in disease outcome. In light of the shared inflammatory component of these diseases, we hypothesize that interfering with the pool of extracellular MT may confer therapeutic or protective effects in early-onset T1D.

## Results

### MT is expressed by pancreatic endocrine cells, most notably in autoantibody-positive patients

**Fig. 1:**
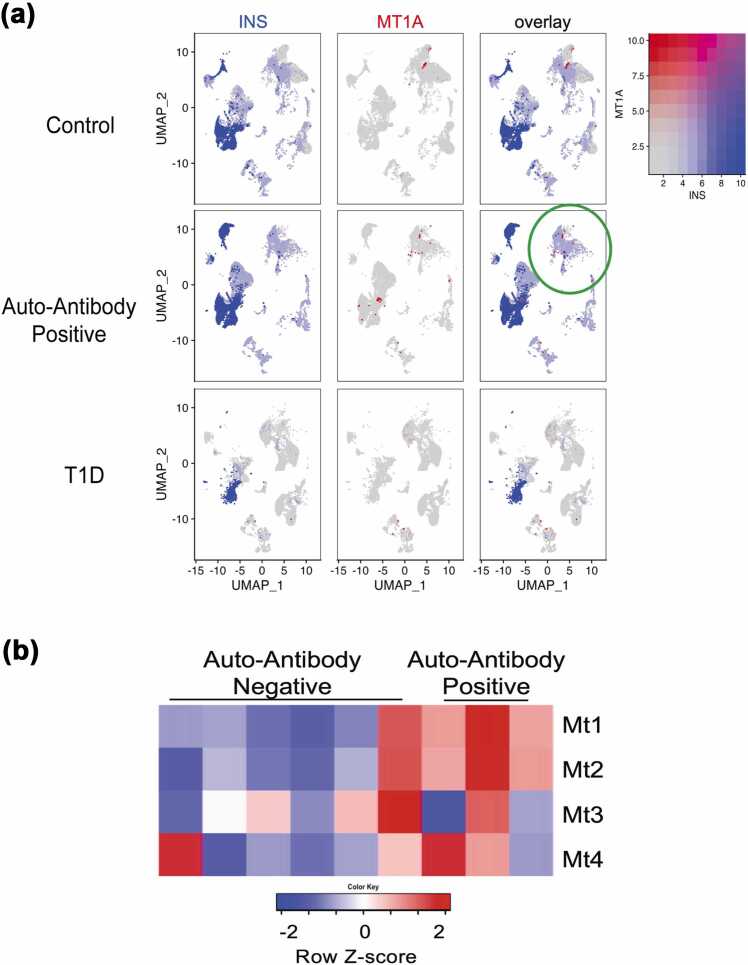
MT is expressed by pancreatic endocrine cells, most notably in autoantibody positive (AAB+) patients. (a) Uniform Manifold Approximation and Projection (UMAP) of mRNA expression in ∼80,000 cells obtained from the pancreatic islets of control (top row) (n = 11), AAB+ (middle row) (n = 8), and type 1 diabetic patients (bottom row) (n = 5). In each set of UMAP plots, the first column shows insulin (*INS*) expression in blue to identify beta cells, the second column shows *MT1A* expression in red and the third column combines expression of *INS* and *MT1A*. The intensity of the color corresponds to the amount of gene expression. Circle indicates expression of both *MT1A* and *INS* in group of cells.^29^ (b) Heatmap of *Mt1*, *Mt2*, *Mt3*, and *Mt4* expression in the pancreatic lymph nodes of AAB+ and AAB− 5-week old nonobese diabetic (NOD) mice.^30^ Abbreviation used: MT, metallothionein.

Since MT has been implicated in other autoimmune and chronic inflammatory diseases, we examined the literature for evidence of MT upregulation in T1D. Several studies have noted the involvement of MT in the progression of T1D with more than one suggestion as to its potential role in beta cell function and disease pathogenesis.^25^, ^26^, ^27^, ^28^ A recent publication reported single-cell transcriptomics from pancreatic islets of established T1D patients, patients with autoantibodies toward pancreatic islet proteins but no clinical diagnosis of T1D, and nondiabetic organ donors with neither autoantibodies nor a history of T1D.^29^ We acquired and reanalyzed this publicly available dataset to investigate *Mt* gene expression in these patients. The highest *Mt1A* gene expression was noted in the islets from prediabetic, autoantibody-positive donors (Figure 1(a)) and several other Mt isoforms were also upregulated in the prediabetic pancreatic cells (Figure S1). Additionally, a report of global gene expression analysis in the pancreas of prediabetic NOD mice revealed increased expression of three isoforms of MT (*Mt1*, *Mt2*, and *Mt3*) compared to congenic control animals,^30^ as summarized in Figure 1(b). The increase in *Mt* gene expression in both humans and NOD mice in pre-onset and early-onset T1D is intriguing and indicates that an important function may occur early in the progression of T1D.

### Min6 beta cells upregulate and release MT when exposed to stress

**Fig. 2:**
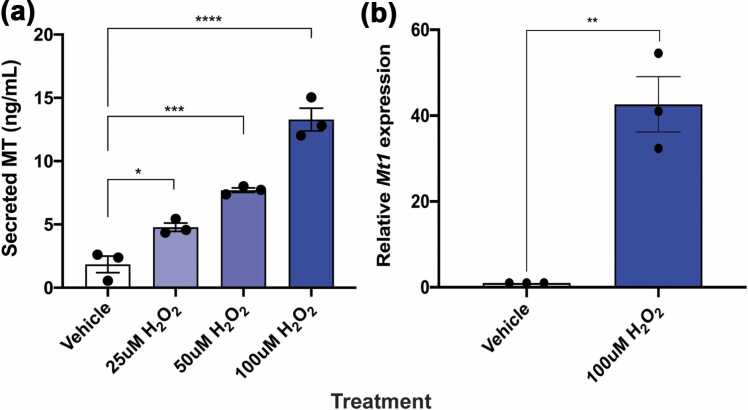
Beta cells release MT when exposed to stress. (a) Min6 cells (mouse pancreatic beta cell line) were plated at 10^6^ cells/mL and exposed to varying concentrations of H_2_O_2_ for 24 h at 37 °C with 5% CO_2_. Supernatant was harvested and cells and debris removed by centrifugation. Cells were harvested and MT in supernatant was quantified by sandwich ELISA. Cell culture media was used as vehicle. Data presented as mean + SEM and are inclusive of three separate experiments. *P < 0.05, ***P < 0.001, ****P < 0.0001 by two-way ANOVA. (b) Min6 cells were plated at 10^6^ cells/mL for 24 h and subsequently exposed to media + 100 μM H_2_O_2_ for 4 h. Cell culture media was used as vehicle. RNA was extracted and cDNA synthesized for analysis by RT-PCR. Results obtained by real-time PCR were processed according to the delta-delta Ct method, using 2^−ΔΔCt^ to determine the final level gene expression normalized against the endogenous control (peptidylprolyl isomerase A), and refer to the value of calibrator (unstimulated cells). Data presented as means + SEM and are inclusive of three separate experiments. **P < 0.01 by ratio paired t test. Abbreviations used: ANOVA, analysis of variance; MT, metallothionein; PCR, polymerase chain reaction; RT-PCR, real-time polymerase chain reaction; SEM, standard error of the mean.

The intracellular functions of MT are well-documented, but its mechanism of release into the extracellular environment is as yet unknown. Its lack of a signal peptide suggests that it exits cells by non-classical pathways, as do some other proteins with immunoregulatory effects, such as IL-1, S100A, FGF-1, and HMGB family proteins.^31^, ^32^, ^33^ We have verified the selective release of MT from leukocytes by exposing cells to a subtoxic dose of cadmium chloride, a potent inducer of MT expression.^34^ Secretion of MT *via* non-classical pathways is supported by *in silico* analysis of MT amino acid sequence using SecretomeP, a program that predicts the likelihood that a protein lacking a signal peptide will nonetheless have a structural profile similar to traditionally secreted extracellular proteins.^35^ To determine if beta cells release MT under stress, we used Min6 cells, a mouse pancreatic beta cell line whose phenotype reflects that of normal islets.^36^ Due to this cell line’s documented ability to lose its glucose responsiveness over time,^37^ we first confirmed a beta cell phenotype by measuring glucose-stimulated insulin secretion (Figure S2(a)). As oxidative stress has been implicated in beta cell dysregulation in early T1D,^38^ we used hydrogen peroxide as the stressor of Min6 cells. After exposure to several non-toxic concentrations of H_2_O_2_ for 24 h, we observed MT release into the supernatant in a dose-dependent manner (Figure 2(a)) without a significant reduction in cell viability (Figure S2(b)). Furthermore, this release of MT coincided with increase in *Mt* gene expression after H_2_O_2_ exposure (Figure 2(b)).

### Extracellular MT influences CXCR4+ Th2 cell chemotaxis

**Fig. 3:**
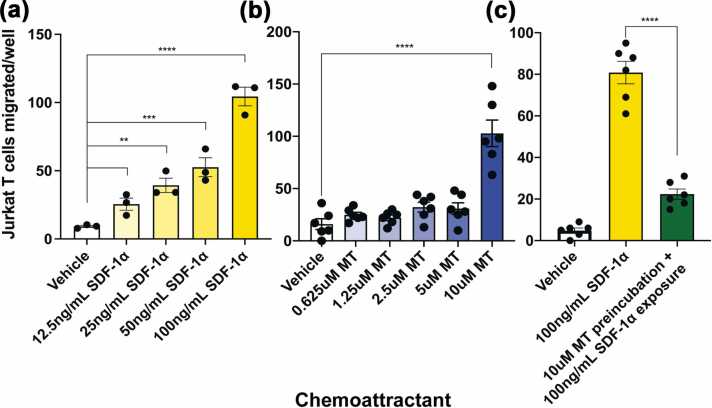
MT is a weak chemoattractant for Th2 cells, but can also inhibit Th2 cell chemotaxis toward SDF-1α. SDF-1α (a) and MT (b) are chemoattractant for Jurkat T cells. Jurkat T cells at 2 × 10^6^ cells/mL were added to the upper wells of the Boyden chamber and exposed to a gradient of SDF-1α (a) or MT (b) and incubated for 3 h at 37 °C with 5% CO_2_. Cells that migrated through the 5 μM pore membrane were fixed and stained and enumerated using a microscope. (c) Exposure to MT can prevent Jurkat T cell chemotaxis to SDF-1α. Jurkat T cells at 2 × 10^6^ cells/mL were exposed to 10uM MT for 1 h at 37 °C with 5% CO_2_, washed, and added to the upper wells of the Boyden chamber before exposure to 100 ng/mL SDF-1α for 3 h at 37 °C with 5% CO_2_. Cells that migrated through the 5 μM pore membrane were stained and enumerated using a microscope. Cell culture media was used as vehicle in all experiments. Data presented as means + SEM and are inclusive (a) or representative (b, c) of three separate experiments. **P < 0.01, ***P < 0.001, ****P < 0.0001 by one-way ANOVA. Abbreviations used: ANOVA, analysis of variance; MT, metallothionein; SEM, standard error of the mean.

SDF-1α (also known as CXCL12) is a well-studied alpha chemokine that is strongly chemotactic for lymphocytes *via* the CXCR4 receptor. In previous studies, CXCR4+ T cells recruited by SDF-1α prevented the development of T1D in an adoptive cell transfer of diabetes.^8^ SDF-1α may also have protective effects in T1D due to AKT phosphorylation downstream of CXCR4 activation, which in turn increases survival and inhibits dedifferentiation of beta cells.^39^, ^40^ We observed a potent chemotactic effect of SDF-1α on CXCR4+ Jurkat T cells (Figure 3(a)). Once released into the extracellular environment, MT acts as a chemoattractant for a number of cell lines and primary cells.^41^ Its 20 cysteine residues among its 61 total amino acids are arranged in CC, CXC, and CX_3_C motifs, equivalent to the motifs used to classify chemotactic cytokine families. Furthermore, there is a syntenic region in both the mouse and human genomes that includes both the MT gene cluster and other chemokine genes. To confirm that Zn_7_-MT can induce chemotaxis, Jurkat T cells were exposed to several concentrations of MT, and cell migration was measured. Migration was induced with the highest concentration of MT (Figure 3(b)). We then tested if MT has the ability to block the chemotactic effects of SDF-1α on these cells and found that preincubation of cells with MT inhibited the effect of SDF-1α on cell migration (Figure 3(c)), suggesting that MT may alter the movement of protective CXCR4+ T cells in T1D. Consistent with this model, we observed that cell incubation with CXCR4 antagonist AMD3100 interferes with MT-mediated Jurkat T cell chemotaxis (Figure S3).

### Extracellular MT influences CXCR3+ Th1 cell chemotaxis

**Fig. 4:**
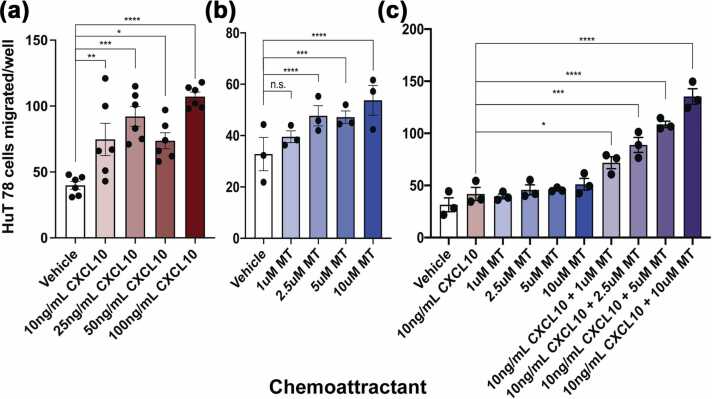
MT can synergistically affect Th1 cell chemotaxis toward CXCL10. CXCL10 (a) and MT (b) are chemoattractant for HuT 78 cells. HuT 78 cells at 1 × 10^6^ cells/mL were serum starved for 4 h, added to the upper wells of the Boyden chamber and exposed to a gradient of CXCL10 (a) or MT (b) and incubated for 3 h at 37 °C with 5% CO_2_. Cells that migrated through the 8 μM pore membrane were fixed and stained and enumerated using a microscope. (c) Co-exposure to MT enhanced HuT 78 cell chemotaxis to substimulatory levels of CXCL10. HuT 78 cells at 1 × 10^6^ cells/mL were serum starved for 4 h, added to the upper wells of the Boyden chamber, and exposed to 10 ng/mL CXCL10+ a gradient of MT 3 h at 37 °C with 5% CO_2_. Cells that passed through the 8 μM pore membrane were fixed and stained and enumerated using a microscope. Cell culture media was used as vehicle in all experiments. Data presented as means + SEM and are representative (a) or inclusive (b, c) of three separate experiments. *P < 0.05, **P < 0.01, ***P < 0.001, ****P < 0.0001 by two way ANOVA. Abbreviations used: ANOVA, analysis of variance; MT, metallothionein; SEM, standard error of the mean.

Another well-documented chemokine receptor-ligand pathway in T1D is the interaction between CXCR3 and CXCL10. CXCL10 is a proinflammatory interferon-γ inducible chemokine whose receptor, CXCR3, is found on Th1 cells.^42^ CXCL10 expression is upregulated in both pancreatic alpha and beta cells in islets of both humans and NOD mice at the onset of T1D.^43^ Once released into the extracellular environment, CXCL10 recruits autoaggressive CXCR3+ lymphocytes to islets that lead to the destruction of beta cells.^44^ The presence of these Th1 cells leads to a positive feedback loop of proinflammatory cytokine release through the secretion of IFNγ and TNFα, which stimulate beta cells to produce more chemokines, thereby potentiating further autoreactive Th1 cell recruitment.^42^ It is important to note that while CXCL10 expression remains elevated in patients with established T1D, it is at its highest levels in new-onset and early-stage T1D.^45^ Blocking CXCL10/CXCR3 with neutralizing antibodies reduces T1D incidence, and T1D is milder in CXCL10/CXCR3 knockout mice, while disease progression is accelerated in animals overexpressing CXCL10.^7^, ^46^, ^47^, ^48^, ^49^ When exposed to a gradient of CXCL10, CXCR3+ HuT 78 cells demonstrate a predictable chemotactic response (Figure 4(a)). Given that MT can act as a chemoattractant for other cell types, we exposed HuT 78 cells to a gradient of MT and observed a mild chemotactic effect (Figure 4(b)). However, when a range of MT concentrations was added to a constant amount of CXCL10 below the concentration needed to evoke a strong chemotactic response, there was a substantial increase in the chemotactic response of the cells (Figure 4(c)). Notably, this same synergistic effect is not observed when exposing Jurkat T cells to a combination of SDF-1α and MT (Figure S4). This additive effect of MT and CXCL10 suggests another important mechanism by which MT may alter the inflammatory environment of early onset T1D, by shifting the chemotactic response from Th2 to Th1 cell infiltration into the islets.

### Anti-MT can block MT’s effects on Th1 and Th2 cell chemotaxis

**Fig. 5:**
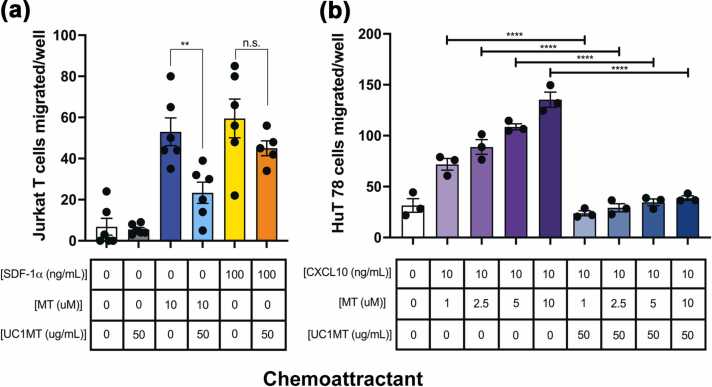
UC1MT binds to MT and blocks its effects on chemotaxis of Th1 and Th2 cells. (a) UC1MT blocks MT-mediated chemotaxis, but not SDF-1α-mediated chemotaxis of Jurkat T cells. Jurkat T cells at 2 × 10^6^ cells/mL were added to the upper wells of the Boyden chamber, and exposed to 10 μM MT or 100 ng/mL SDF-1α + 50 μg/mL UC1MT for 3 h at 37 °C with 5% CO_2_. Cells that migrated through the 5 μM pore membrane were fixed and stained and enumerated using a microscope. Data presented as mean + SEM and representative of three separate experiments. *P* < 0.01 by one way ANOVA. (b) UC1MT blocks the synergistic effect of MT on CXCL10-mediated chemotaxis of HuT 78 cells. HuT 78 cells at 1 × 10^6^/mL were serum starved for 4 h, added to the upper wells of the Boyden chamber, and exposed to 10 ng/mL CXCL10 and a gradient of MT + 50 μg/mL UC1MT for 3 h at 37 °C with 5% CO_2_. Cells that migrated through the 8 μM pore membrane were fixed and stained and enumerated using a microscope. Cell culture media was used as vehicle in all experiments. Data presented as means + SEM and are inclusive of three separate experiments. *P* < 0.0001 by two way ANOVA. Abbreviations used: ANOVA, analysis of variance; MT, metallothionein; SEM, standard error of the mean.

UC1MT is a monoclonal anti-MT antibody that demonstrates highly specific binding to mammalian MT1 and MT2^16^ and has been used to manipulate the *in vivo* pool of MT in several applications. Mice treated with UC1MT exhibit enhanced T-dependent immunity by inhibiting the suppressive effects of both exogenous and/or endogenous MT.^12^, ^16^ UC1MT has also proven effective as a therapeutic treatment when administered in murine models of IBD and AILI, where it interferes with leukocyte infiltration and inflammation.^20^, ^24^ To determine if UC1MT can block the effects of MT on Th1 and Th2 cell chemotaxis toward their cognate chemokines, Jurkat T cells were exposed to SDF-1α or MT in the presence or absence of UC1MT. We found that preincubation of MT with UC1MT was sufficient to block the chemotactic effect of MT on Jurkat cells, but the antibody did not exert the same effect on SDF-1α mediated chemotaxis (Figure 5(a)). Similarly, when HuT 78 cells were exposed to the same concentrations of MT+CXCL10 as in Figure 4(c) with UC1MT present, the synergistic chemotactic effect was ameliorated (Figure 5(b)). These results suggest that UC1MT may confer protective effects by preventing chemotactic dysregulation by MT in inflammation.

### UC1MT reduces inflammation and prevents diabetes in NOD mice

**Fig. 6:**
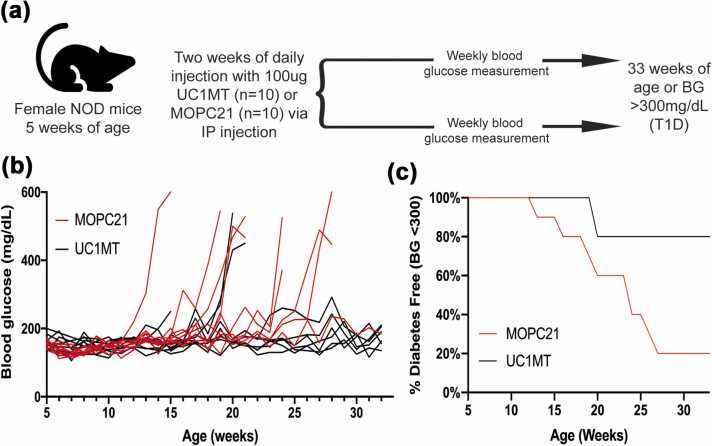
UC1MT prevents diabetes in female NOD mice. To test the therapeutic effect of UC1MT in a model of T1D, NOD female mice were treated with either UC1MT or isotype control IgG MOPC21. Beginning at 5 weeks of age, mice were treated daily for 2 weeks with intraperitoneal injections of 100 μg of UC1MT (n = 10) or MOPC (n = 10) and blood glucose was monitored weekly. Mice were euthanized after onset of T1D (blood glucose > 300 mg/dL). (a) Schematic of cohort 1 experimental timeline. (b) Blood glucose measurements were taken until 33 weeks or age or onset of T1D. (c) Mice treated with UC1MT were protected from hyperglycemia compared to mice treated with MOPC (*P* < 0.01 by log-rank test). Abbreviations used: IP, intraperitoneal; NOD, nonobese diabetic; T1D, type 1 diabetes.

To evaluate the therapeutic utility of UC1MT in a mouse model of insulitis-induced diabetes, NOD female mice were treated with either UC1MT (n = 10) or an isotype-matched control monoclonal IgG_1_, MOPC21 (n = 10) (Figure 6(a)). Starting from 5 weeks of age, mice were treated daily for 2 weeks with intraperitoneal (IP) injections of antibodies and blood glucose levels were monitored until 33 weeks of age or the development of T1D (blood glucose >300 mg/dL) (Figure 6(b)). Mice treated with UC1MT were protected from hyperglycemia when compared to those treated with isotype control antibody (*P* < 0.01 by log-rank test) (Figure 6(c)). Notably, the 2-week UC1MT treatment conferred protection from T1D development until 33 weeks of age, much past the typical onset of T1D in female NOD mice.^50^

**Fig. 7:**
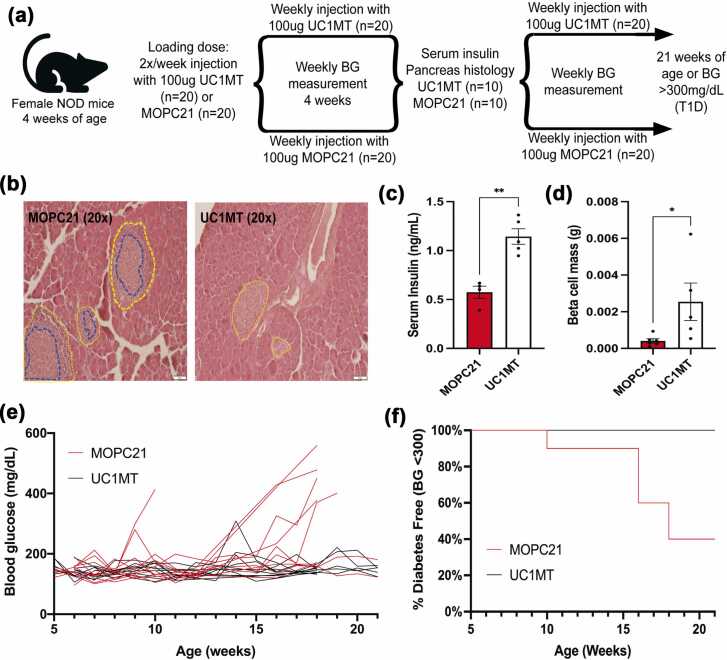
UC1MT reduces inflammation and preserves insulin production in NOD mice. In cohorts 2 and 3, mice were treated 2× in week 4 and 1×/week subsequently with 100 μg of UC1MT (n = 20) or MOPC21 (n = 20). (a) Schematic of cohort 2 and 3 experimental timeline. (b) Lymphocyte infiltration (indicated in blue in the MOPC21-treated pancreas) surrounds the healthy islet tissue (indicated in yellow in both treatment groups); this was blocked by UC1MT by 4 weeks after treatment initiation. Scale bar represents 50 µm. (c) Treatment with UC1MT prevented the decrease of circulating insulin levels. Data presented as means + SEM. **P < 0.01 by Student’s t test. (d) Beta cell mass in mice treated with UC1MT vs MOPC21. Data presented as means + SEM. *P<0.05 by Student's t test. (e) Blood glucose was monitored weekly, and (f) mice treated with UC1MT were again protected from hyperglycemia compared to the control group (P < 0.01 by log-rank test). Abbreviations used: NOD, nonobese diabetic; T1D, type 1 diabetes.

To further evaluate UC1MT treatment during the onset of T1D, another treatment regimen was tested in two independent, replicate cohorts. At 4 weeks of age, mice were administered two injections of UC1MT (n = 10) or MOPC21 (n = 10) to quickly establish a steady-state dose, and subsequently were injected once weekly until euthanasia. Half of the mice were euthanized at 9 weeks, and pancreatic tissue was prepared for histological evaluation (Figure 7(a)). Mice treated with UC1MT showed less leukocyte infiltration around pancreatic islets when compared to isotype control (Figures 7(b) and S5). Additionally, serum insulin was measured and treatment with UC1MT was found to prevent the decrease of circulating insulin levels that are indicative of pancreatic failure (Figure 7(c)). Beta cell mass was also significantly higher in animals treated with UC1MT (Figure 7(d)). The rest of the mice in these cohorts were monitored for blood glucose levels until 21 weeks of age or development of T1D (Figure 7(e)). Mice in the UC1MT treatment group were protected from hyperglycemia and T1D compared to those in the control group at 21 weeks of age (*P* < 0.01 by log-rank test) (Figure 7(f)).

In a separate study to evaluate the safety of UC1MT treatment, we treated C57BL/6 mice with UC1MT (n = 5) or MOPC21 (n = 5) weekly for 5 weeks. We saw that there was no difference in body weight (Figure S6(a)), serum Ig levels (Figure S6(b)), or immune cell populations in the mesenteric lymph nodes (Figure S7(a) and (b)), spleen (Figure S7(c) and (d)), or thymus (Figure S7(e) and (f)). Additionally, the inhibitory effects of UC1MT are specifically related to its ability to bind MT, as no binding was observed between UC1MT and SDF-1α or CXCL10, or any other chemokines included in initial testing aimed at identifying any potential unintended binding interactions (Figure S8). Taken together, these data suggest that UC1MT may be a safe and effective treatment for T1D.

## Discussion

The rapid increase in diagnosed cases of T1D in recent years illuminates an urgent need for early interventions that may help prolong beta cell survival in these patients. We have previously reported that anti-MT monoclonal therapeutic treatment can ameliorate the destructive inflammation of IBD, leading us to evaluate this course of treatment in another model of autoimmune inflammation. We found that MT expression is upregulated in insulin-producing cells early in the progression of T1D, and this MT can be released by beta cells in response to stress. Once in the extracellular environment, it can influence chemotaxis of immune cells by dysregulating the response of both CXCR3+ Th1 cells and CXCR4+ Th2 cells to their cognate chemokines. This effect can be blocked with the treatment of the UC1MT antibody, resulting in a marked decrease in diabetes incidence in NOD mice, less leukocyte infiltration into pancreatic islets, and increased levels of circulating insulin.

Autoantibodies against beta cell antigens including GAD 65 (GADA), zinc transporter 8 (ZnT8A), and insulin (IAA) are frequently the earliest established signs of pancreatic islet autoimmunity. Patient populations with a family history of autoimmune disease, genetic loci associated with susceptibility to T1D (e.g. specific combinations of *HLA-DRB1, -DQA1*, and *-DQB1* alleles), or environmental triggers such as heavy metal exposure or certain viral infections are at higher risk for development of disease-associated auto-antibodies. The presence of these antibodies is a strong predictor for the subsequent development of T1D and marks a critical window for intervention.^51^, ^52^, ^53^ In recent-onset T1D up to 1 year after diagnosis, children (0–14) retain 38% of their insulin-positive, functional islets, with young adults (15–39) preserving 56%. At more than 1 year post-diagnosis, however, that number drops to about 13%.^54^ By administering UC1MT treatment early in the progression of the disease (after the detection of IAA but before further loss of functional beta cell mass), there is a possibility that beta cell function may be retained.

It is striking that in our first cohort, a treatment course of only 2 weeks was enough to prevent the development of diabetes for the subsequent 26 weeks in an animal model that is predicted to have a diabetic incidence of approximately 80% by 25 weeks of age.^55^ It may be that daily treatment at a dose of 4 mg/kg may have provided a high enough accumulation of circulating antibodies to continue MT blockade for the duration of the experimental timeline, given that the half-life of UC1MT in mouse serum as previously tested is about 5.8 days (Figure S9). It may also be that we administered treatment at a crucial window before the onset of overt T1D, and this was able to provide sustained protection against the future development of the disease. In our second treatment regimen, weekly injections of antibody at the same dose were again able to prevent the onset of T1D in all mice of the UC1MT treatment group for the duration of the experimental timeline, suggesting that keeping the amount of circulating antibody relatively stable confers the same benefit.

There are several limitations to the proposed work. The prediabetic window in predisposed patients may be the best time for prophylactic treatment, but it may prove difficult to identify this window accurately and treat this population prophylactically, rather than treating therapeutically after T1D has been clinically identified. One possibility that has been explored in recent years is islet or whole pancreas transplantation. While this method has met with little success thus far, it does provide an exact time course during which treatment should be administered. Interfering with the immunoregulatory effects of MT prior to transplantation may lead to higher rates of success.

Treatments for T1D that show promise in the NOD model do not always translate into successful clinical therapeutics in the human population.^54^ However, there has been some progress made in biological therapy for T1D in humans, such as anti-CD3 monoclonal therapeutics. One potential use for UC1MT in the treatment of established T1D would be in conjunction with anti-CD3 treatment as part of a combination therapy, as suggested by Lasch *et al.*^48^ and Christen and Kimmel.^45^ Monotherapy with anti-CD3 agonizts causes a temporary halt in T1D progression by inactivating helper T cells and allowing for the expansion of regulatory T-cell populations. Teplizumab, a humanized anti-CD3 monoclonal antibody, can delay the onset of stage 3 T1D in at-risk patients, supporting the notion that early treatment can decrease the risk of disease progression.^56^ Other anti-CD3 therapeutics have been met with some success in human trials, but they did not confer lasting protection.^57^, ^58^ Several combination therapies have been tested, typically involving treatment with an anti-CD3 and an immunomodulatory agent such as IL-1RA,^59^ anti-CD20,^60^ or nasal proinsulin.^61^ While these treatments were more successful than anti-CD3 alone, they did not prevent the migration of regenerated autoaggressive T cells into the islets after initial depletion.

Co-therapy with anti-CD3 and anti-CXCL10 showed promising results in animal models of T1D, by preventing some immune cell infiltration into the islets, but neutralization of one chemokine alone may not be sufficient in a disease where many chemokines have overlapping functions.^48^, ^62^ By administering anti-MT treatment along with anti-CD3, autoreactive T cells would be diminished, and T cell development would resume in an environment less rich in MT and its proinflammatory effects. Blocking not just one chemokine, but a promiscuous protein such as MT that may have myriad effects in the islet inflammatory environment is a more dynamic approach to this combination treatment.

Another important factor to consider is that MT has many intracellular functions in the context of diabetes and physiological homeostasis. In fact, the overall body of data regarding MT in diabetes suggests that in addition to its proinflammatory signaling, it may also confer some protective benefits. In a streptozotocin-induced diabetes model, MT was induced in the pancreas using zinc, and these rats demonstrated lower glucose levels and a decrease in the activity of oxygen free radicals.^27^ Indeed, pancreatic islets contain very low levels of intrinsic antioxidant enzymes and are highly susceptible to hyperglycemia-induced oxidative damage, which suggests that MT may play an important role in its capacity as a scavenger of reactive oxygen metabolites.^25^, ^28^ By preventing this increase in oxidative stress, MT also exerts anti-apoptotic and anti-inflammatory effects, through inactivation of MAPK signaling pathways and downregulation of mitochondrial apoptotic molecules.^25^ It is important to note that UC1MT treatment only blocks the extracellular pool of MT, while cells retain the intracellular MT and the protection it confers against oxidative stress.

While the underlying molecular mechanisms of disease pathogenesis are different in IBD, AILI, and T1D, the fact that UC1MT demonstrates therapeutic value in these diseases suggests they may share some common features, such as T cell-mediated inflammation. Given the wide variety of conditions that can induce MT expression, it may be that the same environmental triggers of disease development also induce MT upregulation and subsequent ill effects. For example, exposure to environmental toxicants, including metals, has been shown to increase the incidence of T1D. Cadmium chloride is a potent inducer of MT and can elicit MT release from cells even at concentrations that do not significantly decrease cell viability (Figure S10). While the evidence linking CdCl_2_ to increases in T1D is inconsistent,^63^ these observations do suggest that there may be environmental toxicants that will predispose patients to more severe forms of diabetes as a result of MT induction and release.^64^, ^65^ Taken together, these results suggest that UC1MT may provide therapeutic value by interfering in the earliest stages of T1D and preventing the immune cell infiltration that leads to beta cell destruction and development of T1D.

## Materials and methods

### Cell culture

Min6 cells (courtesy of Nathan Alder, University of Connecticut) were maintained in Dulbecco's Modified Eagle Medium (DMEM) (ThermoFisher Scientific Catalog #11-995-065) supplemented with 10% fetal bovine serum (FBS; R&D Systems Catalog #S11550) and antibiotic–antimycotic (ThermoFisher Scientific Catalog #15240062) at 37 °C with 5% CO_2_. Jurkat T cells (ATCC TIB-152) were maintained in Roswell Park Memorial Institute (RPMI) medium (ATCC Catalog #30-2001) supplemented with 10% FBS (R&D Systems Catalog #S11550) and penicillin–streptomycin (ThermoFisher Scientific Catalog #15070063) at 37 °C with 5% CO_2_. HuT 78 cells (ATCC TIB-161) were maintained in Iscove's Modified Dulbecco's Medium (IMDM) (ATCC Catalog #30-2005) supplemented with 20% FBS (R&D Systems Catalog #S11550) and 1× penicillin–streptomycin (ThermoFisher Scientific Catalog #15070063) at 37 °C with 5% CO_2_.

### Human pancreatic islet single-cell RNA sequencing analysis

Publicly available human pancreatic islet tissue single-cell RNA sequencing data^29^ were analyzed. Briefly, Seurat .rds object was retrieved from CZ CELLxGENE data repository (https://cellxgene.cziscience.com/collections/51544e44–293b-4c2b-8c26–560678423380). FeaturePlot() function from Seurat v4.1.1^66^ was used to generate dual-gene Uniform Manifold Approximation and Projection feature plots. To save plots, ggsave() function from ggplot2 v3.3.6 was utilized.^67^

### Heatmap of Mt gene expression in prediabetic NOD mice

Publicly available mouse pancreatic islet microarray data (Gene Expression Omnibus Series GSE15582) were analyzed. Briefly, gene expression values were placed on a log_2_ scale, and the probe sets encoding MT genes were plotted in a heatmap using the “heatmap.2” function in the “gplots“ package and color palettes from the “RColorBrewer“ package. The probesets' values were centered to have mean zero and restricted to the interval (−2, 2) to aid visualization. All microarray analyses were done in the R programming language (http://www.r-project.org).

### Min6 MT induction

Min6 cells were plated at 10^6^ cells/mL in media containing different concentrations of H_2_O_2_ (100 μM, 50 μM, 25 μM) (ThermoFisher Catalog #BP2633500) for 18 h at 37 °C with 5% CO_2_. Supernatants were harvested, and cells were retained for further analysis.

#### Extraction of total RNA and reverse transcriptase reaction

Cells incubated in media containing 100 μM H_2_O_2_ were harvested after 4 h. Cells incubated in media without H_2_O_2_ were used as the control. Acridine Orange/propidium iodide (Nexcelom Bioscience Catalog #CS2-0106) viability staining and analysis using a Cellometer (Nexcelom Bioscience) was performed to determine the toxicity of H_2_O_2_ doses used to induce MT. Total RNA was isolated from cell pellets with the use of MasterPure Complete DNA and RNA Purification Kit (LGC, Biosearch Technologies Catalog #MC85200) according to the manufacturer’s protocol. The concentration and purity of RNA was determined based on spectrophotometric measurements at 260 nm and 280 nm that were made using a NanoDrop (ThermoFisher Scientific). Reverse transcriptase reaction was carried out on RNA isolated from cell pellets, using the iScript gDNA Clear cDNA Synthesis Kit (Bio-Rad Catalog #1725034) according to the manufacturer’s instructions.

#### Real-time polymerase chain reaction (PCR) analysis

Real-time PCR reactions were carried out with iTaq Universal SYBR Green Supermix (Bio-Rad Catalog #1725120), which included polymerase and reaction buffer, and SYBR Green assay for *Mt1* gene (Bio-Rad Assay #qMmuCED0003677). Reactions were conducted in 96 well plates with CFX96 Real-Time System apparatus from Bio-Rad. Peptidylprolyl isomerase A (*ppia*) was used as an endogenous control (Bio-Rad Assay #qMmuCED0041303). Cells that had not been exposed to H_2_O_2_ were used as the calibrator. Results obtained by real-time PCR were processed using 2^−ΔΔCt^ to determine the final level gene expression normalized against the endogenous control (*ppia*) and refer to the value of the calibrator (unstimulated cells).

#### MT1 ELISA

The supernatant was tested by sandwich ELISA. Immulon 2HB 96 well plates (ThermoFisher Scientific Catalog #1424561) were coated with 4 μg/mL UC1MT anti-MT antibody (ThermoFisher custom production of endotoxin-free antibody from hybridoma cells generated in-house^16^) and incubated overnight at room temperature (RT). Plates were washed and blocked with 2% bovine serum albumin (ThermoFisher Scientific Catalog #AAJ6573122), followed by another wash step and incubation with purified MT1 standards (Enzo Life Sciences Catalog #ALX-202-072-M001) or cell supernatants for 2 h at RT. Following another wash step, plates were incubated with biotinylated UC1MT antibody (antibody biotinylated using ThermoFisher Scientific Catalog #90407) for 2 h at RT. After another wash, streptavidin-HRP (BioLegend Catalog #405103) was added to the wells and incubated in the dark at RT for 20 min. Following a final wash step, 3,3′,5,5′-Tetramethylbenzidine (TMB) substrate (BioLegend Catalog #421501) was added, and color was allowed to develop in the dark at RT for 20 min 2N H_2_SO_4_ was added to stop the color reaction, and optical density (OD_450_) was measured using a Spectramax plate reader (Molecular Devices).

### Measurements of chemotaxis

#### Jurkat chemotaxis

Jurkat T cells were added to the upper wells of the Boyden chamber (Neuro Probe Inc Catalog #AP48) at 2 × 10^6^ cells/mL and exposed to a gradient of SDF-1α (Shenandoah Biotechnology Catalog #100-20) or MT (Enzo Life Sciences Catalog #ALX-202-072-M001) and incubated for 3 h at 37 °C with 5% CO_2_. Cells that passed through the 5 μM pore membrane (Neuro Probe Inc Catalog #PFB5) were fixed and stained using Hema 3 Manual Staining System and Stat Pack (Fisher Scientific Catalog #23-123869) and enumerated using a microscope. To test the effect of MT on cell movement toward SDF-1α, cells were preincubated with MT for 1 h at 37 °C and 5% CO_2_ and washed before being added to the upper wells of the chamber and exposed to a diffusing gradient of 100 ng/mL SDF-1α placed in the lower wells. To evaluate the specificity of MT-mediated chemotaxis, 10 μM MT was preincubated with 50 μg/mL UC1MT for 1 h before addition to the bottom wells, and cells were added to upper wells at 2 × 10^6^ cells/mL.

#### HuT 78 chemotaxis

HuT 78 cells were serum-starved for 4 h and added to the upper wells of the Boyden chamber at 10^6^ cells/mL and exposed to a gradient of CXCL10 (100 ng/mL, 50 ng/mL, 25 ng/mL, 10 ng/mL) (Shenandoah Biotechnology Catalog #100-127), MT (10 μM, 5 μM, 2.5 μM, 1 μM), or MT+CXCL10 for 3 h at 37 °C with 5% CO_2_. Cells that passed through the 8 μM pore membrane (Neuro Probe Inc Catalog #PFB8) were fixed and stained using Hema 3 Manual Staining System and Stat Pack (Fisher Scientific Catalog #23-123869) and enumerated using a microscope. To block the effects of MT on HuT 78 chemotaxis, UC1MT was added to the bottom wells of the Boyden chamber at a concentration of 50 μg/mL in addition to 10 ng/mL CXCL10 and 10 μM, 5 μM, 2.5 μM, or 1 μM MT.

### NOD mouse UC1MT treatment

NOD/ShiLtJ (Strain #001976) mice were purchased from The Jackson Laboratory and maintained under specific pathogen-free conditions. Age-matched 4-week-old or 5-week-old female mice were used in all experiments (due to a higher incidence of T1D with a more rapid and consistent onset in female mice). All mice were acclimated for 1 week in the Joslin Institute vivarium and allowed ad libitum access to water and food.

In cohort 1, beginning at 5 weeks of age, mice were administered 100 μg (4 mg/kg) of UC1MT (ThermoFisher custom production of endotoxin-free antibody from hybridoma cells generated in-house^16^) (n = 10) or MOPC21 (BioLegend Catalog #400192) (n = 10) daily *via* IP injection for 2 weeks. Blood glucose was monitored weekly *via* tail nick as described previously^68^ and mice were euthanized *via* CO_2_ inhalation at the onset of diabetes (BG > 300 mg/dL) or at 33 weeks of age.

In cohorts 2 and 3, at 4 weeks of age, mice were injected IP with 100 μg (4 mg/kg) of UC1MT (n = 10) or MOPC21 (n = 10) twice during the week to quickly reach steady-state dose. Subsequently, mice were treated once per week with 100 μg of UC1MT or MOPC21, and blood glucose was monitored as described previously.^68^ Five mice per group were euthanized *via* CO_2_ inhalation at 9 weeks of age and tissues were harvested for histology and blood was collected for cytokine analysis. For hematoxylin and eosin staining, sections were prepared, processed, and stained as described.^69^ Serum insulin was measured by ELISA (Crystal Chem Inc Catalog #90080). Beta cell mass was estimated by (blinded) morphometrically quantifying the total islet area within at least five separate pancreatic sections of at least 100 mm^2^ for each mouse. This relative area was multiplied by the total weight of the pancreas. Pancreatic sections stained with hematoxylin and eosin were ranked for insulitis by blinded counting using the following grades: 0, normal islet morphology with no periinsulitis or insulitis; 1, periinsulitis; 2, insulitis; and 3, islet remnant. The remaining mice were monitored weekly until 21 weeks of age.

### Statistical analysis

Numbers of animals were determined using power analysis. Mice were assigned at random to treatment groups for all mouse studies using a random number generator. Data were collected and processed randomly. Mice numbers (n) per experimental group are stated in each figure legend. The specific tests used to analyze each set of experiments are indicated in the figure legends. Statistical analyses were performed using Prism (GraphPad Software). Multiple groups were compared using one-way or two-way analysis of variance with Bonferroni’s correction for multiple comparisons. Single comparisons were performed using a two-sided T-test. Log-rank test was used to determine the significance in % diabetes-free analysis. In the graphs, *y-*axis error bars represent the SEM as indicated. No outlier values were excluded.

### Study approval

All animal studies in the present work were performed under institutionally approved protocols for the use of animal research (Joslin Diabetes Center and/or the University of Connecticut Institutional Animal Care and Use Committee).

### Conclusion

These data suggest that MT released during the progression of T1D acts as a proinflammatory danger signal that can be mitigated by a brief course of treatment with a monoclonal anti-metallothion antibody. Similar observations for IBD and AILI may suggest that this is a shared phenomenon amongst disease with a chronic inflammatory component.

## Funding and support

This work was supported by grants from the 10.13039/100000002National Institutes of Health (R01ES007408 to MAL; R01DK077097 to YHT; F32DK102320 and K01DK111714 to MDL, P30DK036836 to Joslin Diabetes Center), the American Diabetes Association (ADA 7-12-BS-191 to YHT), and Biohaven, Ltd. to MAL

## Author contribution

MAL, CKM, MDL conceptualized the work; MAL, CKM, MDL, SB, CMP, AVT, CEN, LL, YHT contributed to the methodology; CKM, MDL, SB, SCS, CMP, AVT, CEN contributed to investigation; CKM, MDL, CMP performed data curation and visualization; MAL, LL, YHT, MDL acquired funding; CKM, MAL wrote original draft; all authors reviewed and edited manuscript. The order of the co-first authors was determined by their efforts and contributions to the manuscript.

## Declarations of interest

The authors declare the following financial interests/personal relationships which may be considered as potential competing interests: Michael A. Lynes reports financial support was provided by the National Institutes of Health, Connecticut Innovations, the Program in Innovative Therapeutics for Connecticut’s Health (PITCH), and Biohaven Ltd. Yu-Hua Tseng reports financial support was provided by National Institutes of Health and the American Diabetes Association. Matthew D. Lynes reports financial support was provided by the National Institutes of Health. Michael A. Lynes reports a relationship with Biohaven Ltd that includes: consulting or advisory and funding grants. Michael A. Lynes has patent #20140141009-A1 “Use of antagonists targeting metallothionein to treat intestinal inflammation” issued to Assignees: University of Connecticut, University of Gent; Licensee: Biohaven Ltd. Michael A. Lynes has patent #20220306731-A1 “Metallothionein antibodies and their use” licensed to Assignee: University of Connecticut; Licensee: Biohaven Ltd. Michael A. Lynes, Yu-Hua Tseng, Matthew D. Lynes have patent # 11,866,488-B2 “Compositions comprising an anti-metallothionein antibody and a pancreatic cell targeting moiety” licensed to Assignees: University of Connecticut, Joslin Diabetes Center, Inc.; Licensee: Biohaven Ltd. If there are other authors, they declare that they have no known competing financial interests or personal relationships that could have appeared to influence the work reported in this paper.

## Data Availability

I have shared the link to my data at the Attach File step. MT and T1D (Original data) (Mendeley Data)
