# Supplementary material for: Extracellular metallothionein as a therapeutic target in the early progression of type 1 diabetes
Source: Cell Stress Chaperones. 2024 Mar 13;29(2):312–25. doi: 10.1016/j.cstres.2024.03.005 (PMC10990868; doi:10.1016/j.cstres.2024.03.005)
Supplement: Supplementary file 1 — Supplementary material [file mmc1.docx]

Supplementary Material

# Supplementary Materials and Methods

**1.1 Glucose Stimulated Insulin Secretion in Min6 Cells**

Min6 cells were plated at 8x10^5^/mL in a 24-well plate and cultured for 3 days at 37C with 5% CO_2_. Prior to the glucose stimulation, cells were starved in Krebs-Ringer buffer (KRB) containing 137mM NaCl, 4.7mM KCl, 2.5mM CaCl_2_, 1.2mM MgSO_4_, 1.2mM KH_2_PO_4_, 25mM NaHCO_3_, 10mM Hepes, 1% bovine serum albumin, and 2.8mM glucose for one hour, then washed three times with KRB. Cells were then incubated in KRB with Hepes and BSA plus 2.8, 16.7, or 25mM glucose for one hour. The supernatants were collected, and the secreted insulin was measured using an insulin ELISA kit according to manufacturers’ directions (Crystal Chem Inc Catalog #90080).

**1.2 Measurements of Chemotaxis**

*1.2.1* *AMD3100 blocking Jurkat T cell chemotaxis*

Jurkat T cells at 2x10^6^/mL were pre-incubated with or without 20uM AMD3100 (Sigma Aldrich Catalog #239820) for 30 minutes at 37C with 5% CO_2_. The cells were then washed and added to the upper wells of the Boyden chamber assay (Neuro Probe Inc Catalog #AP48). MT (10uM) (Enzo Life Sciences Catalog # ALX-202-072-M001) and SDF-1α (12.5nM) (Shenandoah Biotechnology Catalog #100-20) were added to the bottom wells of the chamber as chemoattractants and the chamber was incubated for 3 hours at 37C with 5% CO_2_. Cells that passed through the 5uM pore membrane (Neuro Probe Inc Catalog #PFB5) were fixed and stained using Hema 3^TM^ Manual Staining System and Stat Pack (Fisher Scientific Catalog #23-123869) and enumerated using a microscope.

*1.2.2 SDF-1a and MT co-incubation*

Jurkat T cells at 2x10^6^/mL were added to the upper wells of the Boyden chamber assay (Neuro Probe Inc Catalog #AP48). A range of concentrations of SDF-1α (Shenandoah Biotechnology Catalog #100-20) from 100ng/mL to 12.5ng/mL were pre-incubated with or without 1uM MT (Enzo Life Sciences Catalog # ALX-202-072-M001) for one hour at 37C and then added to the bottom wells of the chamber as chemoattractants and the chamber was incubated for 3 hours at 37C with 5% CO_2_. Cells that passed through the 5uM pore membrane (Neuro Probe Inc Catalog #PFB5) were fixed and stained using Hema 3^TM^ Manual Staining System and Stat Pack (Fisher Scientific Catalog #23-123869) and enumerated using a microscope.

**1.3 Safety Study**

C57BL/6 mice were randomly assigned to treatment groups and injected weekly i.p. with 100ug of UC1MT (n=5) (ThermoFisher custom production of endotoxin-free antibody from hybridoma cells generated in-house (Lynes et al., 1993)) or MOPC21 (n=4) (BioLegend Catalog #400192) for six weeks. Animals were weighed weekly and blood was collected for analysis of total serum immunoglobulin levels. After six weeks, mice were euthanized and blood and tissues harvested for further analysis.

*1.3.1* *Total serum immunoglobulin ELISA*

Immulon 2HB plates (ThermoFisher Scientific Catalog #1424561) were coated with 4ug/mL anti-mouse Ig (Southern Biotech Catalog #1010-01) and incubated overnight at RT. After washing, plates were blocked with 2% BSA (ThermoFisher Scientific Catalog #AAJ6573122) for 2h at RT followed by another wash step. Dilutions of mouse Ig (Biomeda Catalog #MS147) were plated to generate a standard curve, along with dilutions of mouse serum from the study animals and incubated for 2h at RT, followed by a wash, after which alkaline phosphatase conjugated anti-mouse Ig (Southern Biotech Catalog #1010-04) was added to the plate for 2h at RT. Following a final wash step, 1mg/mL para-Nitrophenylphosphate (pNPP) developing buffer (ThermoFisher Scientific Catalog #37621) was added to the wells, and OD_405_ measurements were taken every 30 seconds for ten minutes using a Spectramax plate reader (Molecular Devices).

*1.3.2 Flow cytometry analysis of immune cell populations*

Single cell suspensions were prepared from spleen, lymph nodes (axial, brachial, and mesenteric) and thymus, and red blood cells were lysed with Ammonium-Chloride-Potassium (ACK; 150mM NH_4_Cl, 10mM KCl, 0.1mM Na_2_-EDTA) lysis buffer (spleen) or water (lymph nodes, thymus). Aliquots of each sample were stained with two different antibody cocktails as shown below (Table 1). OneComp eBeads (ThermoFisher Catalog #01111141) were used to prepare compensation controls, and fluorescence minus one (FMO) controls were prepared using cells. Samples were analyzed on a BD Biosciences Fortessa X-20 cytometer. Gating analysis was performed with FlowJo (BD Biosciences), and statistical analysis was done in Prism (GraphPad). The makeup of the antibody panels is shown in supplementary table 1. Panel 1 was used to identify T cell subsets and NK cells, and Panel 2 was used to identify B cells and subsets of myeloid cells. The two markers labeled with APC in Panel 2 are on mutually exclusive sets of cells that can be gated away from each other before visualizing the APC label.

| **Panel 1** |  |  | **Panel 2** |  |
| --- | --- | --- | --- | --- |
| **Marker** | **Label** |  | **Marker** | **Label** |
| CD3 | AlexaFluor 700  BioLegend Catalog #100216 |  | CD19 | Brilliant Violet 785  BioLegend Catalog #115543 |
| CD4 | PerCP-Cy5.5  BioLegend Catalog #100433 |  | CD45R | PerCP-Cy5.5  BioLegend Catalog #103235 |
| CD8 | APC-Fire  BioLegend Catalog #100766 |  | CD69 | FITC  BioLegend Catalog #104505 |
| CD25 | Brilliant Violet 785  BioLegend Catalog #102051 |  | CD22 | APC  BioLegend Catalog #126109 |
| CD62L | APC  BioLegend Catalog #104411 |  | F4/80 | PE  BioLegend Catalog #123110 |
| CD44 | PE  BioLegend Catalog #103023 |  | CD11b | APC  BioLegend Catalog #101211 |
|  | Brilliant Violet 421  BioLegend Catalog #137611 |  | CD68 | Brilliant Violet 421  BioLegend Catalog #137017 |
| NK1.1 | AlexaFluor 488  BioLegend Catalog #108717 |  | Ly6G | Brilliant Violet 711  BioLegend Catalog #127643 |
|  |  |  | Ly6C | APC-Fire  BioLegend Catalog #128046 |

**Table 1**

All samples were initially gated for single, intact cells using scatter gates. For Panel 1, spleen and lymph node samples were gated as follows:

T cells were identified as CD3+ and either CD4+ or CD8+. Both CD4 and CD8 single positive populations were then gated on CD44 and CD62L to differentiate naïve, memory, and effector cells. CD4 single positive cells were also gated on CD25 to identify the T_reg_ population. Natural killer (NK) cells were identified as NK1.1+CD335+. Thymus cells were initially gated on CD4 and CD8. CD4 and CD8 single positive cells were further gated on CD3. Double positive and double negative populations were not gated further. For Panel 2, spleen and lymph node samples were gated as follows:

B cells were identified as CD19+CD45R+CD22+CD69-. CD19- F4/80- Ly6G+ cells were tentatively identified as neutrophils based on surface markers and scatter characteristics. Further gating of CD19- F4/80+/- Ly6G- cells is shown in Table 2.

| Parent gate |  |  |  |  |  |
| --- | --- | --- | --- | --- | --- |
| F4/80- | CD11b+ Ly6C- | CD11b+ Ly6C+ | CD11b- Ly6C hi | CD11b- Ly6C int* |  |
|  |  |  |  |  |  |
| F4/80+ | CD11b hi  Ly6C hi | CD11b hi Ly6C int | CD11b+  Ly6C- | CD11b lo  Ly6C- | CD11b-  Ly6C+ |

*intermediate

**Table 2**

Apart from the CD11b^hi^ Ly6C^int^ population (identified as eosinophils based on scatter), the sub-populations of F4/80+ and F4/80- cells were not identified as specific cell types. The only population measured with Panel 2 in samples from thymus was a minor population of CD19+CD45R+ cells.

**1.4 Chemokine ELISAs**

Duoset ELISA kits were used to measure both chemokine-specific and UC1MT antibody binding to SDF-1a, CXCL10, IL-8, CCL2, CCL5, and CCL17 (R&D Systems Catalog #s DY350, DY266, DY208, DY279, DY278, and DY364, respectively). Immulon 2HB plates (ThermoFisher Scientific Catalog #1424561) were coated with 1ug/mL of either UC1MT (ThermoFisher custom production of endotoxin-free antibody from hybridoma cells generated in-house (Lynes et al., 1993)) or anti- SDF-1a, CXCL10, IL-8, CCL2, CCL5, or CCL17 and incubated overnight at RT. After washing, plates were blocked with 2% BSA (ThermoFisher Scientific Catalog #AAJ6573122) for 2h at RT followed by another wash step. Dilutions of chemokine were prepared in a diluent of 1% BSA and incubated on the plate for 2h at RT. After another wash, biotinylated anti-chemokine antibodies prepared according to manufacturer’s directions were added to the plate for 2h at RT. Plates were then washed and incubated with streptavidin-HRP according to manufacturer’s directions for 20 minutes at RT in the dark. After a final wash step, TMB substrate (BioLegend Catalog #421501) was added to the wells and allowed to incubate in the dark at RT for 15 minutes before the addition of 2N H_2_SO_4_ to stop the reaction and OD_450_ measured using a Spectramax plate reader (Molecular Devices).

**1.5 UC1MT Pharmacokinetic Measurements**

C.B-17 SCID mice were obtained from Charles River at 6 weeks of age and injected i.p. with 100ug UC1MT (ThermoFisher custom production of endotoxin-free antibody from hybridoma cells generated in-house (Lynes et al., 1993). Blood samples were taken 3, 7, and 10 days after injection. To measure serum UC1MT, Immulon 2HB plates were coated with 1ug/mL MT (Enzo Life Sciences Catalog # ALX-202-072-M001) overnight at RT. After washing, plates were blocked with 2% BSA for 2h at RT, washed again, and incubated with serum samples for 2h at RT. Following another wash, anti-mouse IgG_1_-HRP (Southern Biotech Catalog #1010-05) was added to the plates and incubated for 2h at RT in the dark. After a final wash, TMB substrate was added to the plates and color was allowed to develop for 15 minutes at RT in the dark. The enzymatic reaction was stopped with the addition of 2N H_2_SO_4_ and OD_450_ was measured using a Spectramax plate reader (Molecular Devices).

**1.6 MT release in CdCl_2_-treated Jurkat T cells**

Jurkat T cells were plated at 10^6^/mL in RPMI (ATCC Catalog #30-2001) containing 20, 10, 5, or 2.5uM CdCl_2_ or vehicle control and incubated for 18h at 37C with 5% CO_2_. Following incubation, supernatants were harvested and cells removed for viability testing via AO/PI (Nexcelom Bioscience Catalog #CS2-0106) staining and Cellometer measurement (Nexcelom Bioscience). MT in supernatants was quantified by sandwich ELISA as described in main text.

# Supplementary Figures


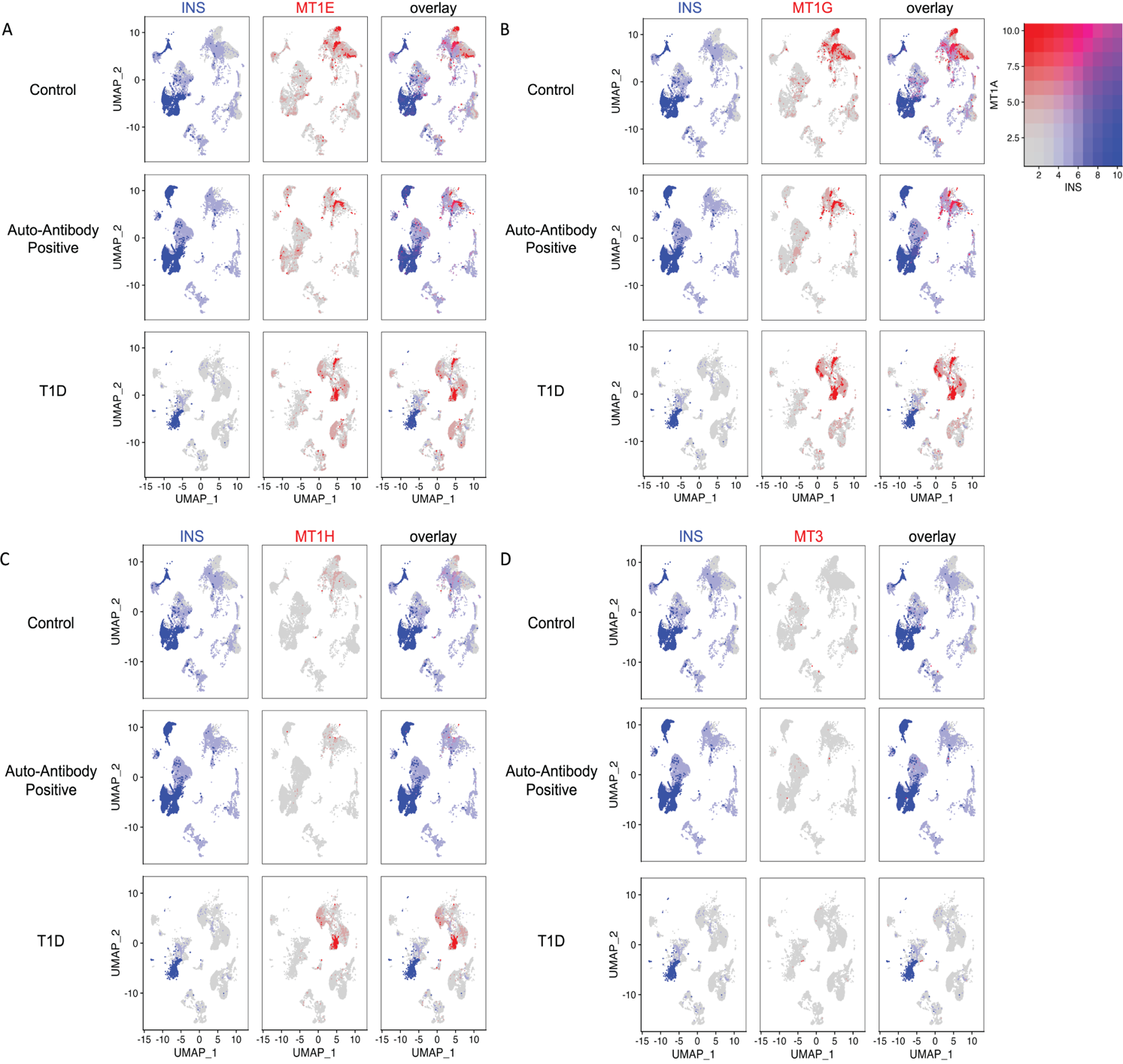


**Supplementary Figure 1.** **Mt gene expression in pancreatic endocrine cells.** Uniform Manifold Approximation and Projection (UMAP) of mRNA expression in ~80,000 cells obtained from the pancreatic islets of control (top row) (n=11), Auto-Antibody (AAB) positive (middle row) (n=8), and Type 1 diabetic patients (bottom row) (n=5). In each set of UMAP plots, the first column shows insulin (*INS*) expression in blue to identify beta cells, the second column shows *MT* isoform expression in red and the third column combines expression of *INS* and *MT*. The intensity of the color corresponds to the amount of gene expression. (A) *MT1E* (B) *MT1G* (C) *MT1H* (D) *MT3.*

**
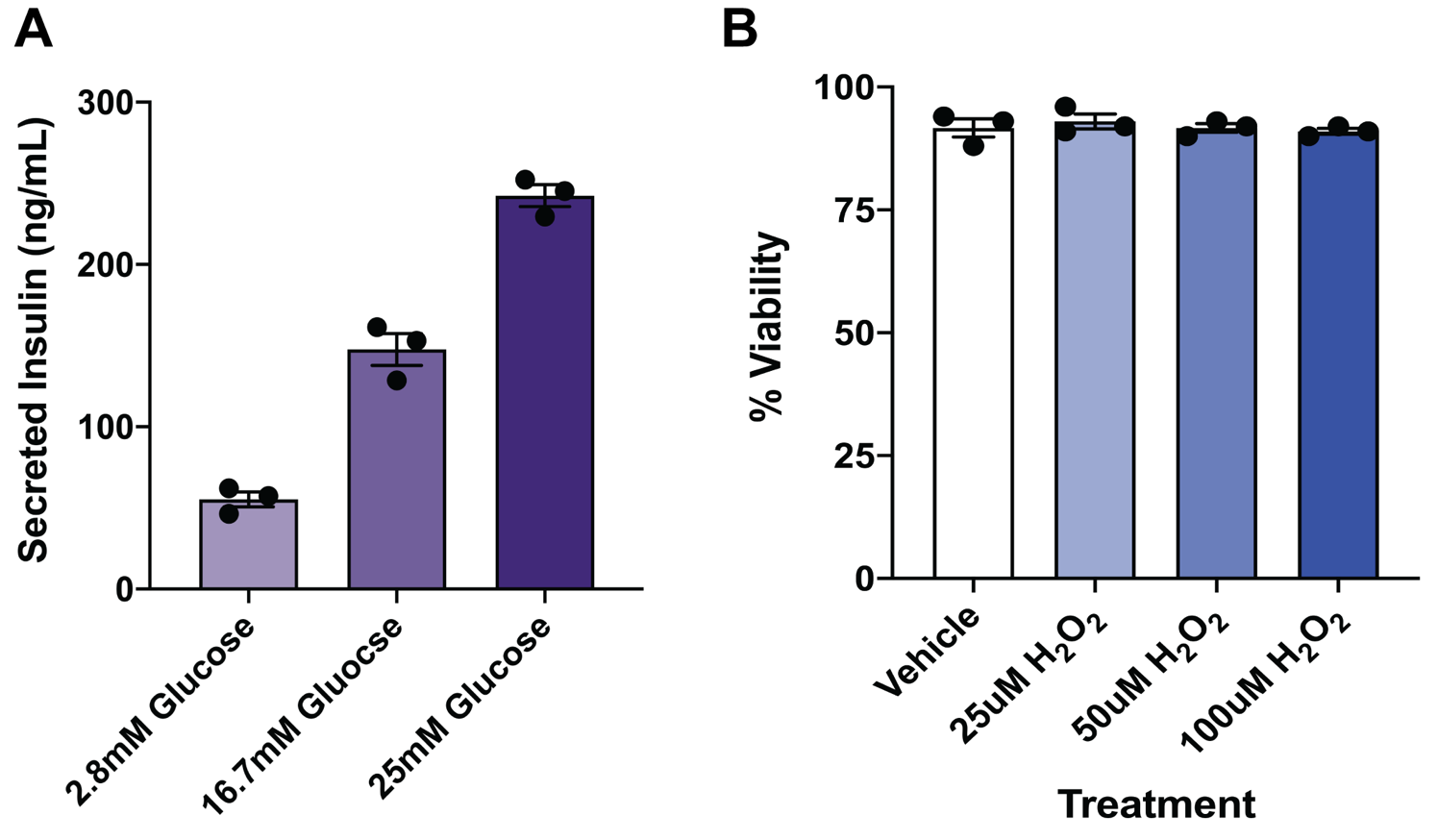
**

**Supplementary Figure 2.** **Min6 cells maintain beta cell phenotype and are viable after H_2_O_2_ exposure.** (A) Min6 were plated at 8x10^5^ cells/mL in a 24-well plate and cultured for 3 days at 37°C with 5% CO_2_. Prior to the glucose stimulation, cells were starved in Krebs-Ringer buffer (KRB) containing 10mM Hepes, 1% bovine serum albumin, and 2.8mM glucose for one hour, then washed three times with KRB. Cells were then incubated in KRB with Hepes and BSA plus 2.8, 16.7, or 25mM glucose for one hour. Insulin was measured by ELISA. (B) Min6 cells were plated at 10^6^ cells/mL and exposed to varying concentrations of H_2_O_2_ for 18 hours at 37°C with 5% CO_2_. Supernatant was harvested and cell viability was measured using Acridine Orange/propidium iodine (AO/PI) staining and analysis by Cellometer instrumentation. Cell culture media was used as vehicle.

**
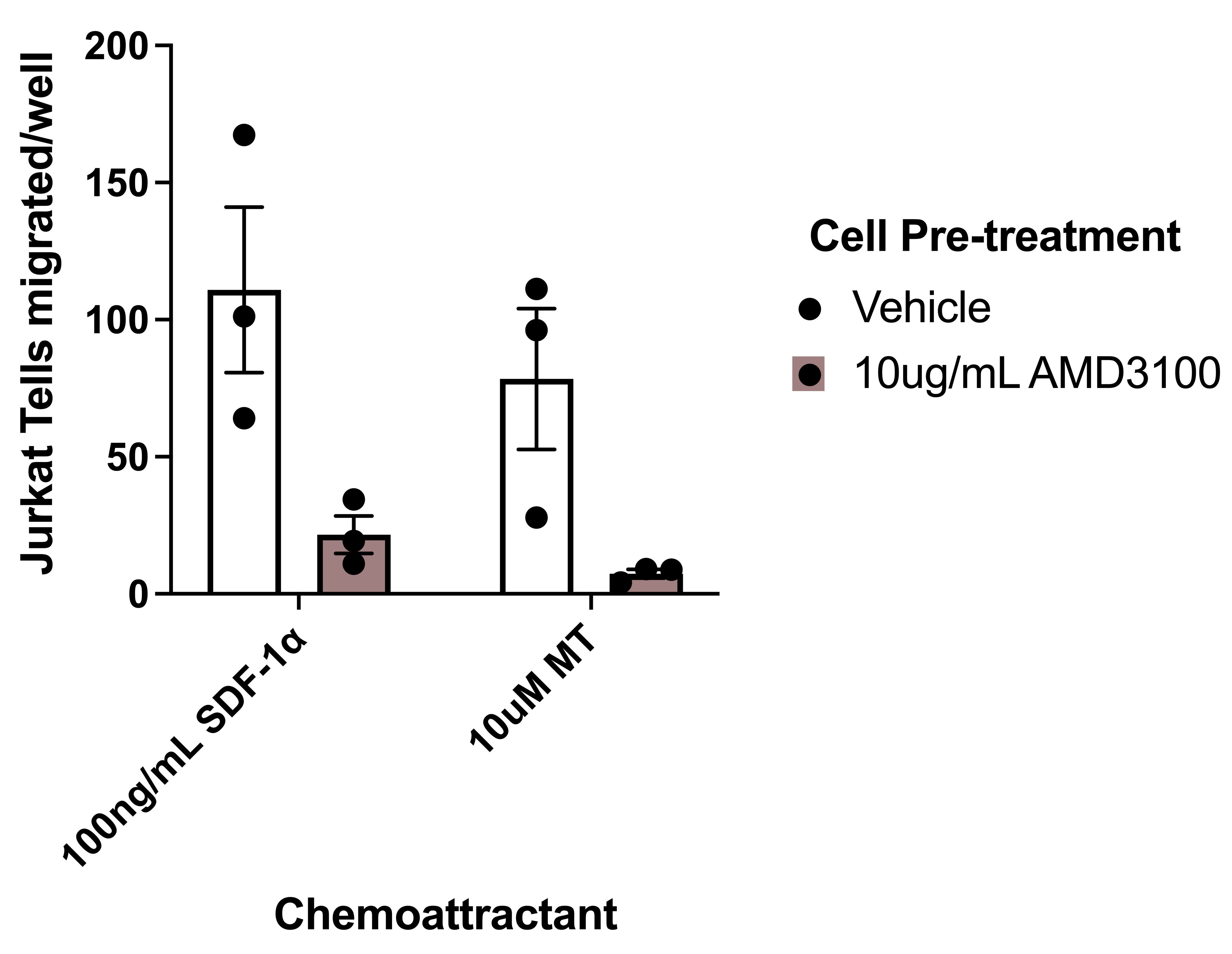
**

**Supplementary Figure 3.** **AMD3100 blocks the MT-mediated chemotactic response.** Jurkat T cells at 2x10^6^ cells/mL were pre-incubated + 10ug/mL AMD3100 for 30 minutes at 37°C with 5% CO_2_. The cells were then washed and added to the upper wells of the Boyden chamber. MT (10uM) and SDF-1α (100ng/mL) were added to the lower wells as chemoattractants and the chamber was incubated for 3 hours at 37°C with 5% CO_2_. Cells that migrated through the 5uM pore membrane were fixed and stained and enumerated using a microscope. Cell culture media was used as vehicle. Data presented as means + SEM and representative of 3 separate experiments. **P<0.01, ***P<0.001 by two-way ANOVA.


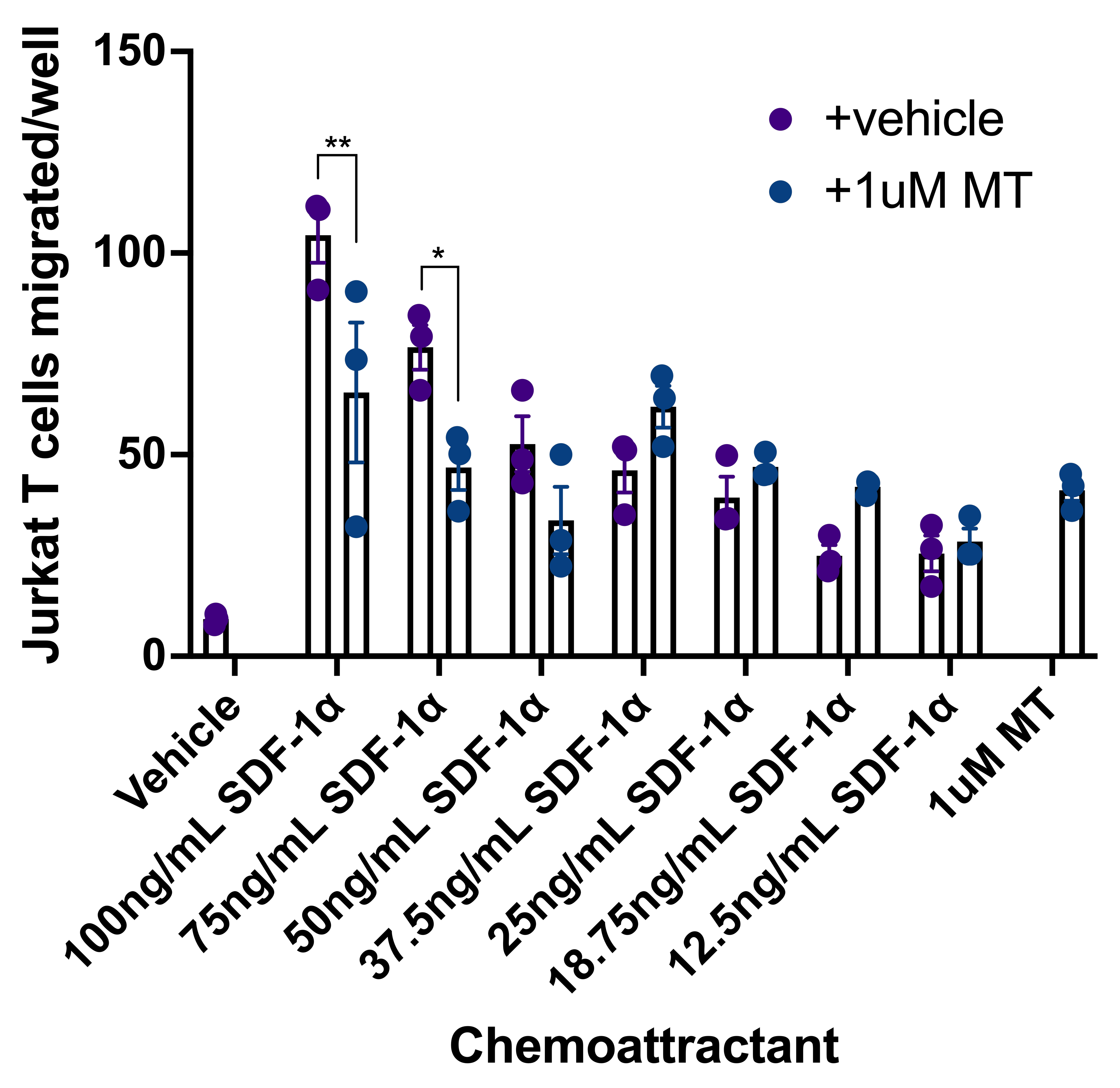


**Supplementary Figure 4.** **MT co-exposure with SDF-1a does not increase Jurkat T cell chemotactic response.** Jurkat T cells were added to the upper wells of the Boyden chamber at 2x10^6^ cells/mL. SDF-1a dilutions + 1uM MT were added to the lower wells as chemoattractants and the chamber was incubated for 3 hours at 37°C with 5% CO2. Cells that migrated through the 5uM pore membrane were fixed and stained and enumerated using a microscope. Cell culture media was used as vehicle. Data presented as means + SEM and are inclusive of three separate experiments. *p<0.05 **p<0.01 by two-way ANOVA.


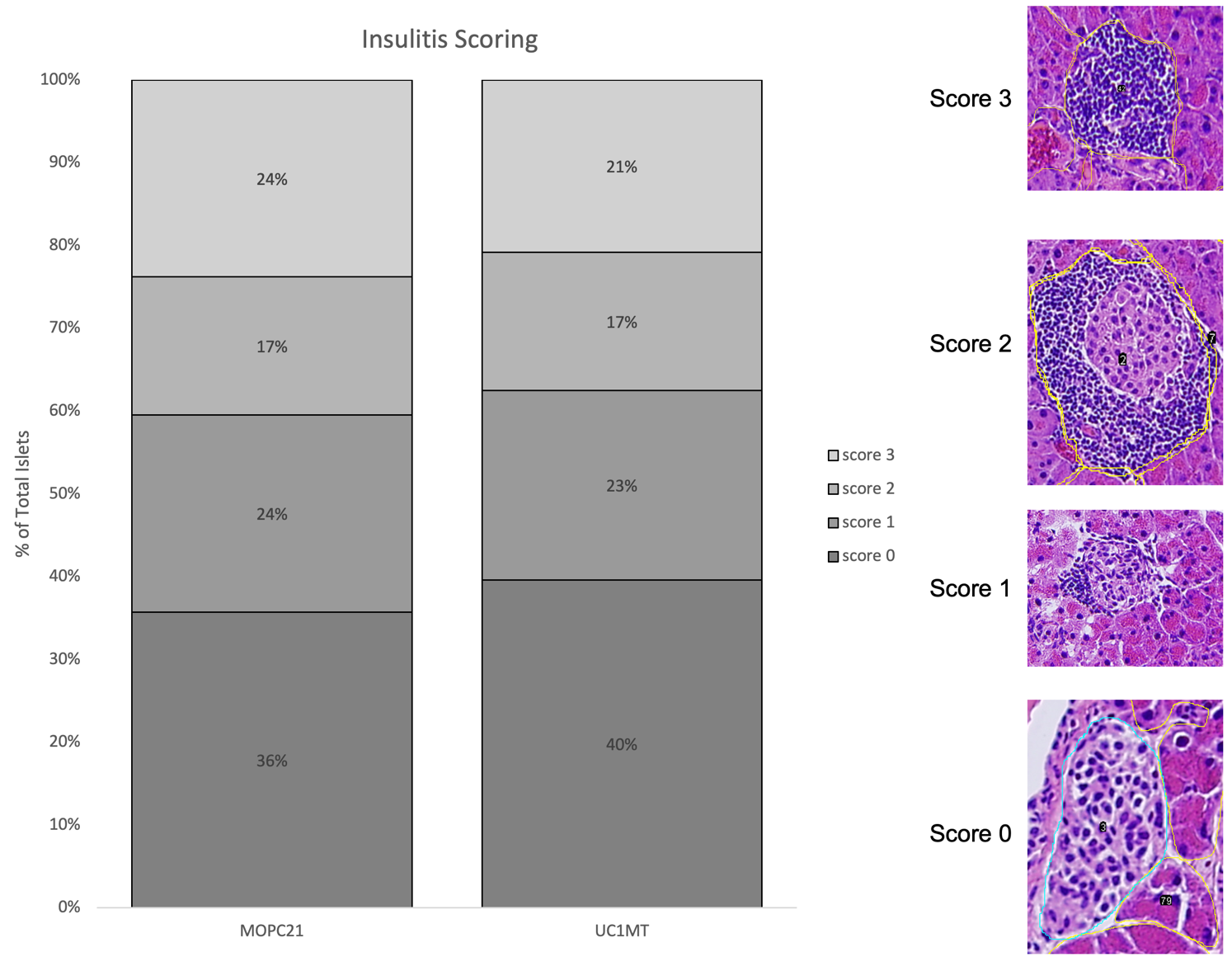
**Supplementary Figure 5**: Percent distribution among insulitis grades for MOPC21 treated mouse islets (n=84) or UC1MT treated mouse islets (96) scored by blinded analysis of hematoxylin and eosin stained pancreatic sections using the following grades: 0, normal islet morphology with no periinsulitis or insulitis; 1, periinsulitis; 2, insulitis; and 3, islet remnant with examples of islets from each grade shown.


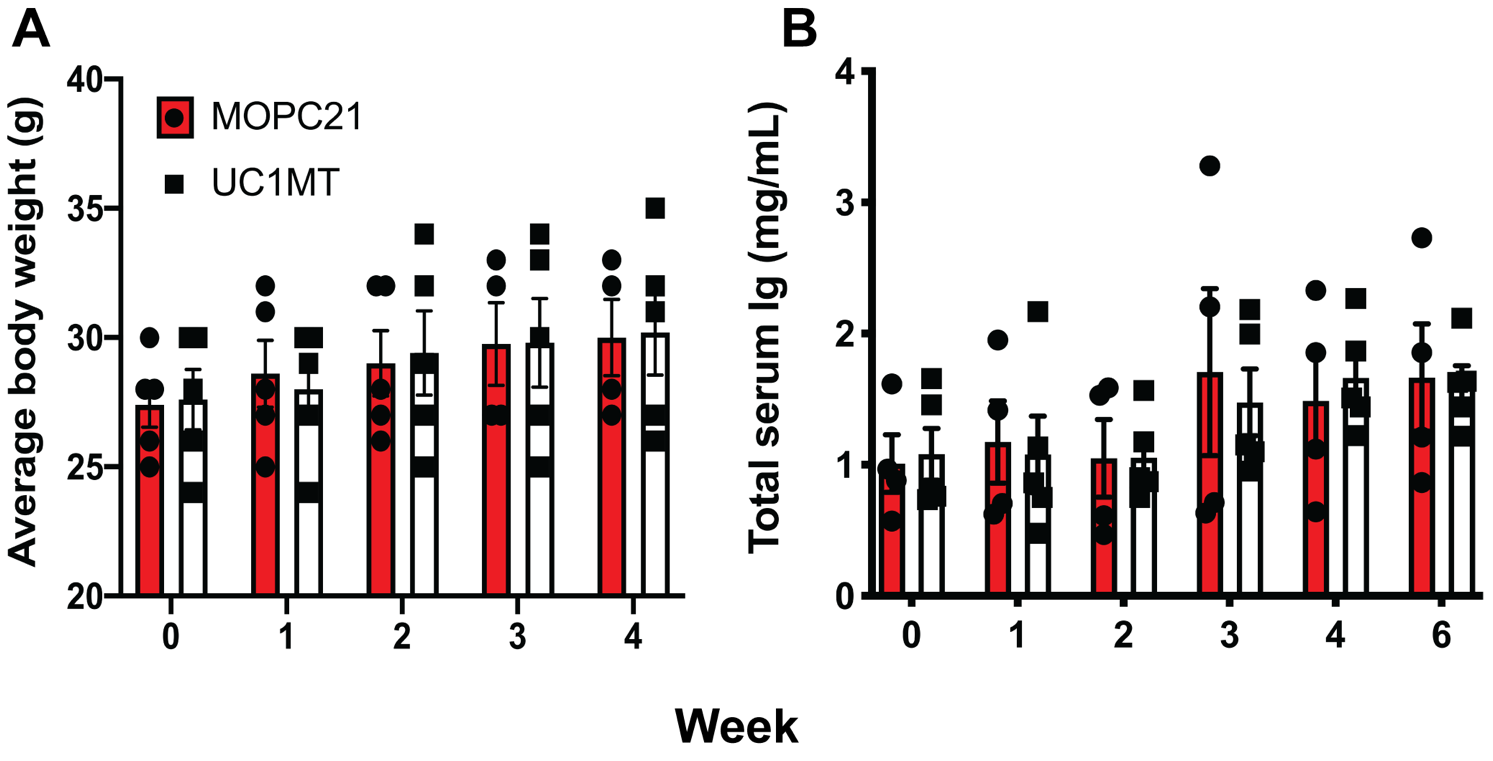


**Supplementary Figure 6.** **UC1MT treatment does not affect body weight or serum Ig.** Age-matched male C57Bl/6J mice were treated with weekly i.p. injections of 100ug UC1MT (n=5) or MOPC21 (n=4) in sterile saline. (A) Animals’ weights were monitored weekly. (B) Blood samples were collected weekly and total immunoglobulin levels were measured by ELISA.


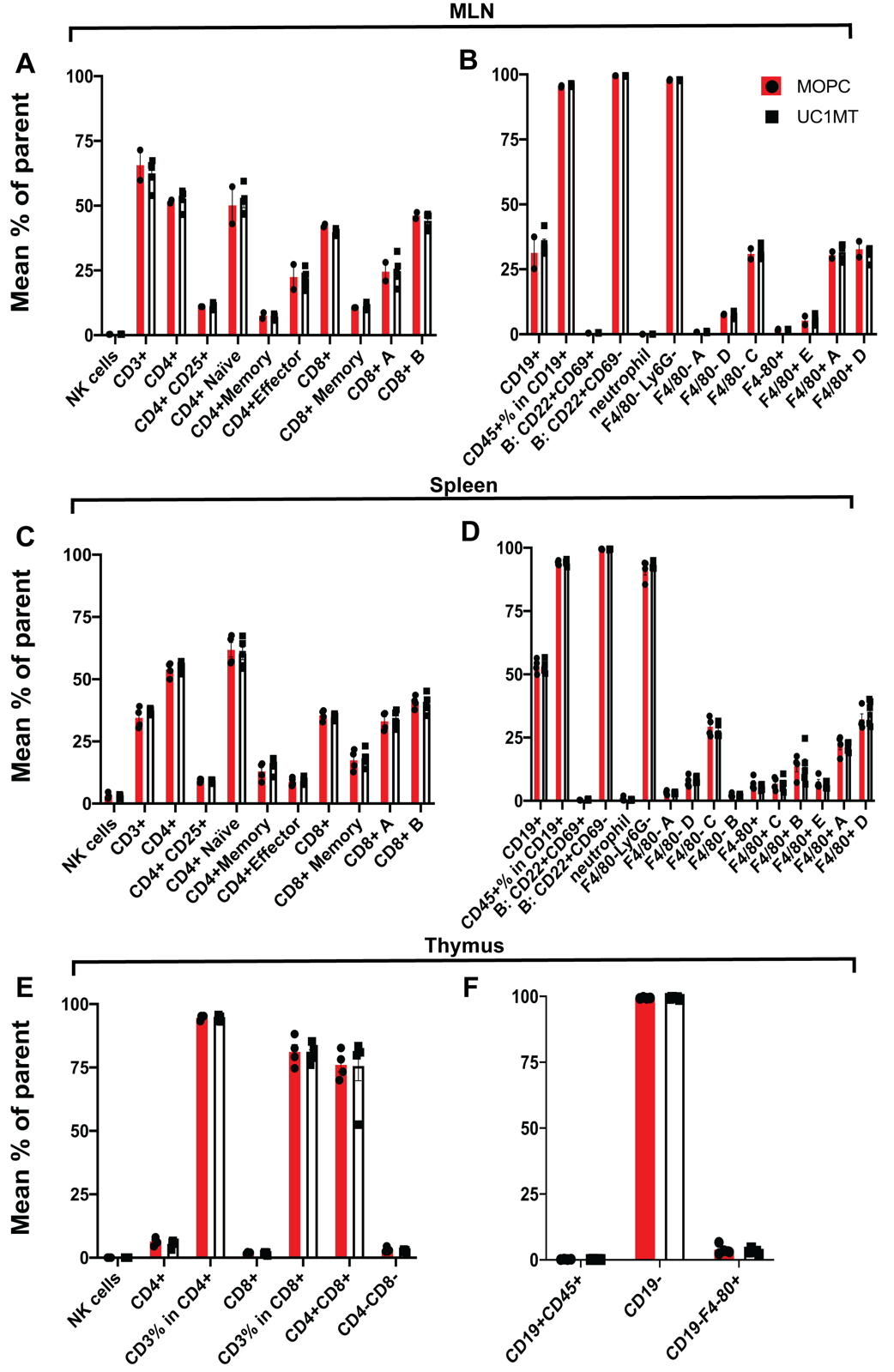


**Supplementary Figure 7.** **UC1MT treatment does not affect immune cell populations in lymphoid tissue.** Age-matched male C57Bl/6J mice were treated with weekly i.p. injections of 100ug UC1MT (n=5) or MOPC21 (n=4) in sterile saline. Animals were euthanized after six weeks and tissues harvested for further analysis. Single cell suspensions were prepared from mesenteric lymph nodes (MLNs) (A,B), spleens (C,D), and thymic tissue (E,F) and red blood cells lysed. Aliquots of each sample were stained with two different antibody cocktails (T/NK cells or B cells/monocytes/granulocytes). OneComp eBeads were used to prepare compensation controls, and FMO controls were prepared using cells. Samples were analyzed on a BD BioSciences Fortessa X-20 cytometer. Gating analysis was performed with FlowJo and statistical analysis was done in Prism.


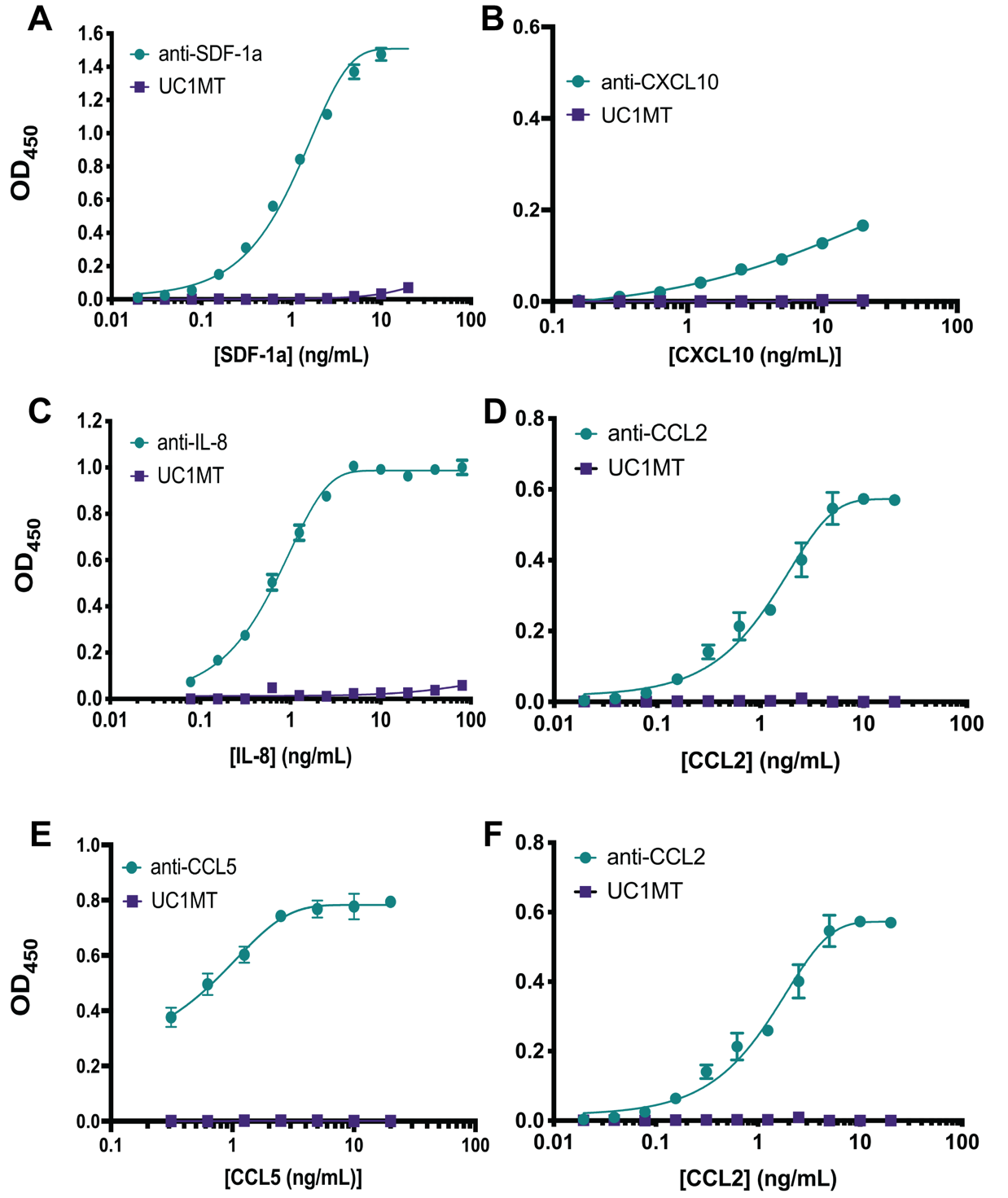


**Supplementary Figure 8.** **UC1MT does not bind to other chemokines.** Immulon 2HB plates were coated with 4ug/mL UC1MT and 4ug anti-SDF-1α (A), anti-CXCL10 (B), anti-IL-8 (C), anti-CCL2 (D), anti-CCL5 (E), or anti-CCL17 (F) overnight at room temperature (RT), washed, and blocked with 2% BSA for 2h at RT. After another wash, dilutions of indicated chemokines in 1% BSA were added to the wells and incubated for 2h at RT. Plates were washed again and incubated with manufacturers’ recommended concentrations of biotinylated anti-SDF-1α (A), anti-CXCL10 (B), anti-IL-8 (C), anti-CCL2 (D), anti-CCL5 (E), or anti-CCL17 (F) for 2h at RT. Following another wash, streptavidin-HRP was added to the plates at manufacturers’ recommended dilution and incubated for 20 min at RT in the dark. After a final wash, TMB substrate was added to the plates and color was allowed to develop for 15 minutes at RT in the dark. The enzymatic reaction was stopped with the addition of 2N H_2_SO_4_ and OD_450_ was measured using a Spectramax plate reader.


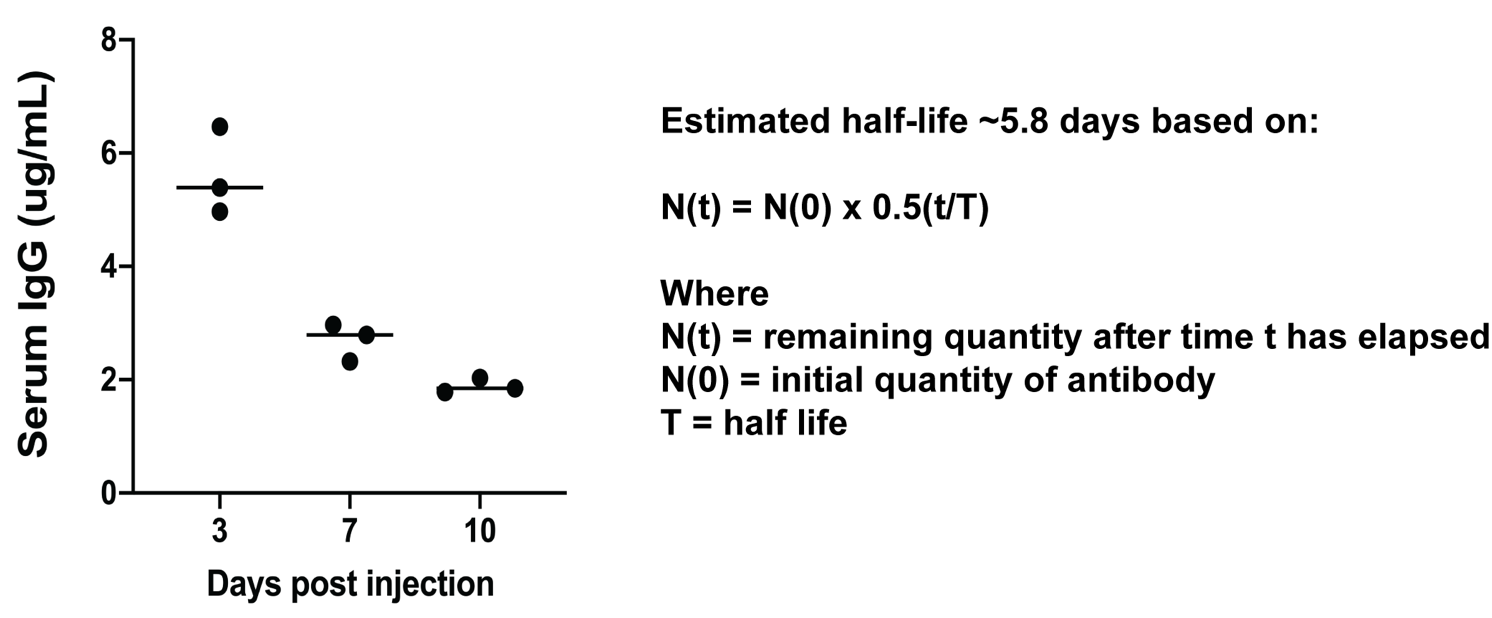


**Supplementary Figure 9.** **UC1MT half-life in mouse serum.** Female C.B-17 SCID mice were obtained from Charles River at 6 weeks of age and injected i.p. with 100ug UC1MT (n=3). Blood samples were taken 3, 7, and 10 days after injection. Serum UC1MT was measured by ELISA.


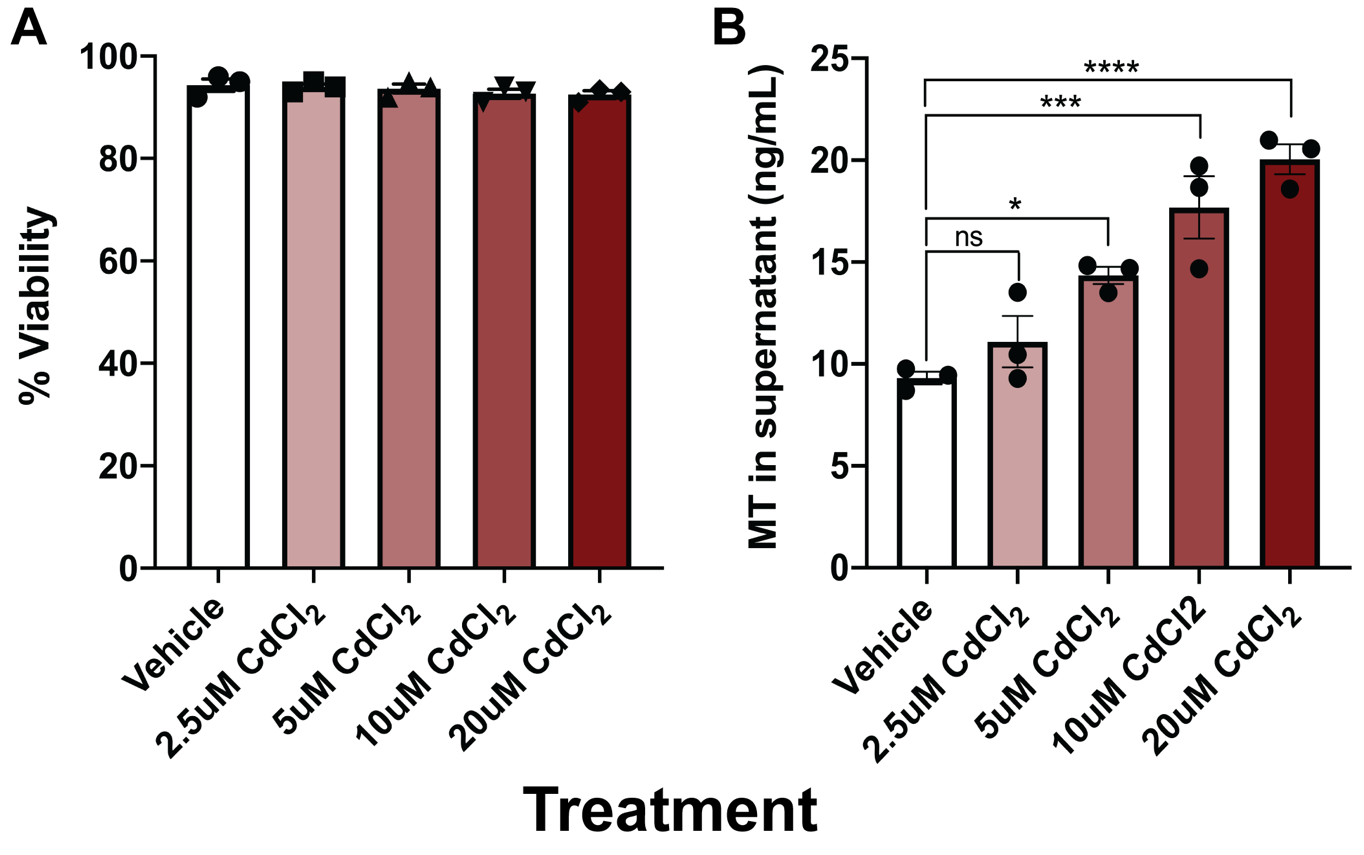


**Supplementary Figure 10.** **CdCl_2_ exposure stimulates Jurkat T cell MT release.** Jurkat T cells were plated at 10^6^ cells/mL in RPMI containing 20, 10, 5, or 2.5uM CdCl_2_ and incubated for 18h at 37°C with 5% CO_2_. (A) Following incubation, supernatants were harvested and cells removed for viability testing via AO/PI staining and Cellometer measurement. (B) MT in supernatants was quantified by sandwich ELISA. Cell culture media was used as vehicle. Data presented as means + SEM and are inclusive of three separate experiments. *P<0.05 ***P<0.001****P<0.0001 by two-way ANOVA.
